# Design, Synthesis, and Evaluation of Linker-Optimised PSMA-Targeting Radioligands

**DOI:** 10.3390/pharmaceutics14051098

**Published:** 2022-05-20

**Authors:** Fanny Lundmark, Gustav Olanders, Sara Sophie Rinne, Ayman Abouzayed, Anna Orlova, Ulrika Rosenström

**Affiliations:** 1 Department of Medicinal Chemistry, Uppsala University, 751 23 Uppsala, Sweden; fanny.lundmark@ilk.uu.se (F.L.); gustav.olanders@ilk.uu.se (G.O.); sara.rinne@ilk.uu.se (S.S.R.); ayman.abouzayed@ilk.uu.se (A.A.); anna.orlova@ilk.uu.se (A.O.); 2 Science for Life Laboratory, Department of Medicinal Chemistry, Uppsala University, 751 23 Uppsala, Sweden

**Keywords:** prostate cancer, PSMA, linker optimisation, molecular imaging, SPPS, structure-based design, PSMA-targeting

## Abstract

Prostate-specific membrane antigen (PSMA) is overexpressed in the majority of prostate cancer cells and is considered to be an important target for the molecular imaging and therapy of prostate cancer. Herein, we present the design, synthesis, and evaluation of 11 PSMA-binding radioligands with modified linker structures, focusing on the relationship between molecular structure and targeting properties. The linker design was based on 2-naphthyl-L-alanine-tranexamic acid, the linker structure of PSMA-617. X-ray crystal-structure analysis of PSMA and structure-based design were used to generate the linker modifications, suggesting that substitution of tranexamic acid could lead to interactions with Phe546, Trp541, and Arg43 within the binding cavity. After synthesis through SPPS, analogues were labelled with indium-111 and evaluated in vitro for their specific binding, affinity, and cellular retention. Selected compounds were further evaluated in vivo in PSMA-expressing tumour-bearing mice. Based on the results, 2-naphthyl-L-alanine appears to be crucial for good targeting properties, whereas tranexamic acid could be replaced by other substituents. [^111^In]In-BQ7859, consisting of a 2-naphthyl-L-alanine-L-tyrosine linker, demonstrated favourable targeting properties. The substitution of tranexamic acid for L-tyrosine in the linker led to an improved tumour-to-blood ratio, highlighting [^111^In]In-BQ7859 as a promising PSMA-targeting radioligand.

## 1. Introduction

Prostate cancer (PCa) continues to be one of the most frequently diagnosed forms of cancer amongst men, and one of the leading causes of cancer-related deaths. For successful treatment, an early diagnosis and correct staging are crucial, as they are integral to treatment planning and patient management. Correct staging is vital for the treatment of patients with distant metastasis, for whom the five-year survival rate drops by 70 percentage points when compared to patients with localized PCa [1]. PCa as diagnosis is currently based on the measurement of prostate-specific antigen (PSA) in blood, digital rectal examinations (DRE), and histopathological verification, together with the use of different imaging modalities such as trans-rectal ultrasound (TRUS), computed tomography (CT), and magnetic resonance imaging (MRI) [2]. However, these methods come with a risk of infection and have limited sensitivity and specificity, especially for the detection of metastasis. Furthermore, these methods do not provide detailed information about the individual molecular characteristics of the cancer, e.g., the expression of potential biomarkers and therapeutic targets. To address this, molecular imaging using positron emission tomography (PET) and single-photon emission computed tomography (SPECT) in combination with CT or MRI have been highlighted as multimodal technologies with high precision and sensitivity. The benefit of simultaneous anatomical and functional data allows for the detection of microscale lesions leading to increased diagnostic accuracy [3,4,5].

PCa cells possess specific pathophysiological characteristics, such as the overexpression of prostate-specific membrane antigen (PSMA), which can be utilized for molecular imaging and targeted radiotherapy. PSMA is a 750 amino acid transmembrane protein consisting of a small intracellular component, a transmembrane domain, and an extensive extracellular segment. It is expressed in the majority of prostate carcinomas and has been detected in both poorly differentiated tumours and metastatic lesions. Benign prostate cells, as well as the small intestine, kidneys, and salivary glands, have an endogenous expression of PSMA. However, the expression is significantly lower (100–1000 fold) than in malignant PCa cells, which makes PSMA an important and highly specific biomarker for PCa [6,7]. The first crystal structure of PSMA was isolated in 2005 and has contributed to a crucial understanding of the protein structure as well as important interactions for potent ligands [8]. The binding cavity of PSMA is a funnel-shaped 20 Å-deep accessory tunnel leading into the active site, which contains two Zn^2+^ ions. Apart from the active site, several subpockets have been identified within the binding cavity. These include the so-called S1′ pharmacophore pocket with a high affinity for glutamate and glutamate-like moieties, the arene-binding site, and the S1 non-pharmacophore pocket, which contains a positively charged “arginine patch” and a “hydrophobic pocket” [9,10].

For PSMA-binding ligands labelled with radiometals, a chelator is necessary to enable the incorporation of a radionuclide. It has been emphasised that a linker is needed to separate the binding moiety from the bulky metal chelator, in combination with keeping it outside the binding cavity. The introduction of a linker also entails additional interactions with subpockets in the binding cavity [8,11]. Structural modifications of the linker have been shown to be more tolerated than changes to the stricter part that targets the active site. This highlights linker modifications as an appealing area when working towards finding more potent PSMA inhibitors with improved pharmacokinetics [11,12]. To alter properties such as affinity, the rate of internalisation, and imaging contrast, the linker needs to adopt a suitable confirmation within the binding cavity to interact with various subpockets that are remote from the active site. Studies have shown that the length, polarity, size, flexibility, the presence of aromatic groups, and the distance of hydrophobic functionality from the active site all play an important role in an optimal linker structure [13,14,15,16,17].

**Figure 1:**
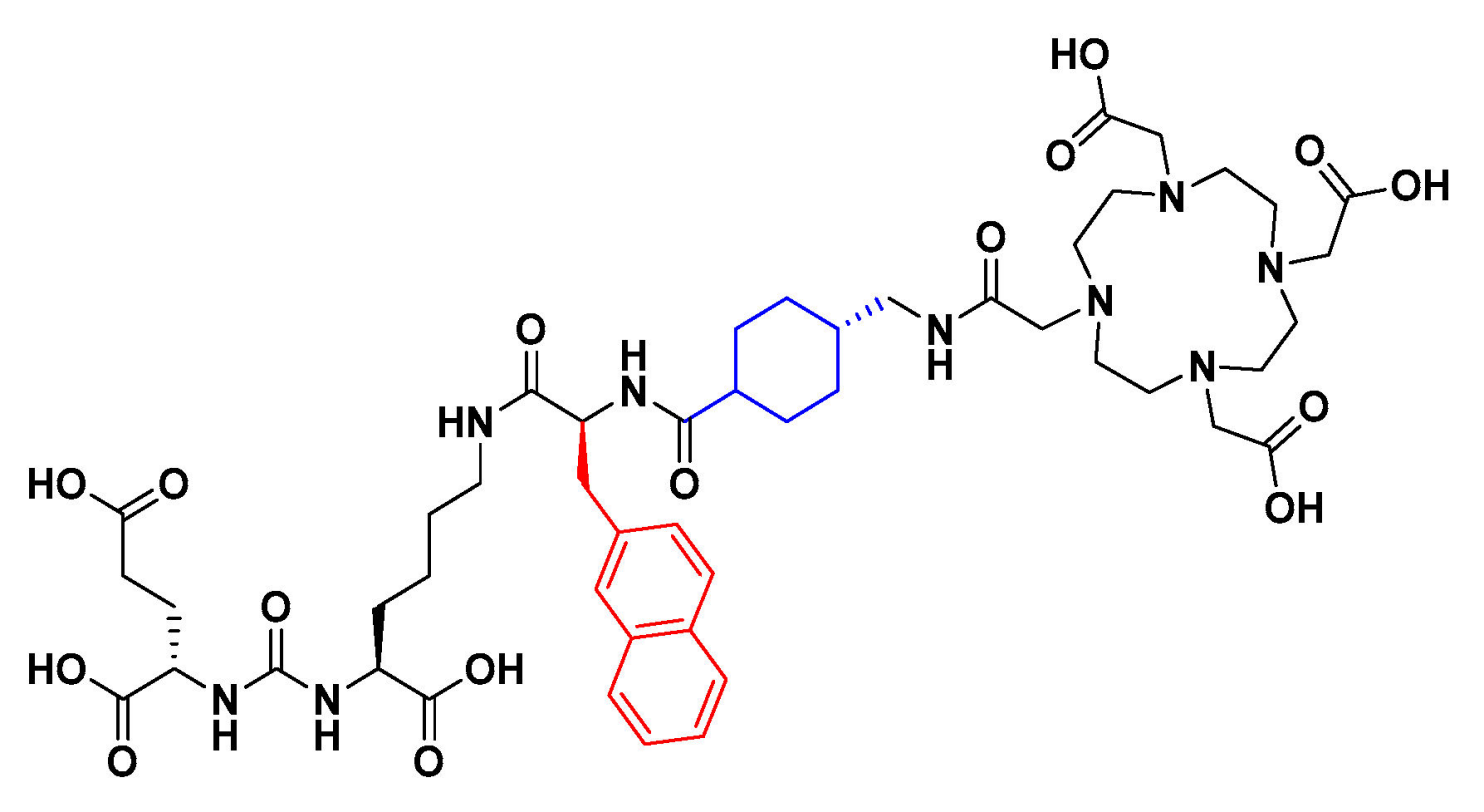
Chemical structure of PSMA-617. The naphthylic linker structure is highlighted in red and the cyclohexyl linker structure in blue.

Several different types of PSMA-targeting inhibitors have been investigated throughout the years. These include hydroxyphosphinyl-, thiol-, and phosphoramidate-based derivatives, all of which have shown high affinity to PSMA. However, the most common and extensively investigated type of high-affinity inhibitors are urea-based ligands, due to both favourable interactions with the active site and synthetic simplicity [10]. The development and clinical evaluation of the urea-based radioligand [^68^Ga]Ga-PSMA-11, constructed of a Glu-urea-Lys binding moiety connected to the HBED-CC radiometal chelator through an aliphatic carbon chain linker, is considered as being a breakthrough in the field [18,19]. Since then, several urea-based PSMA-binding radioligands for both imaging and therapy have been developed [20,21,22]. In 2015, PSMA-617 (Figure 1) was reported, which is constructed of the same urea-based binding moiety as PSMA-11, but with a modified linker consisting of 2-naphthyl-L-alanine and tranexamic acid [23]. PSMA-617 has shown promising targeting properties such as a low kidney uptake, fast background clearance, and high tumour retention. During the last years, [^177^Lu]Lu-PSMA-617 has been involved in several clinical trials for radioligand therapy (RLT) of PCa [21,22] and very recently, [^177^Lu]Lu-PSMA-617 was approved by the FDA as the first targeted RLT for treatment of progressive PSMA-positive metastatic castration-resistant PCa [24]. The improved characteristics of PSMA-617 are thought to be caused by the presence of 2-naphthyl-L-alanine in the linker structure, which favourably interacts with the S1 hydrophobic pocket [10,23]. The linker structure of PSMA-617 has been investigated previously, substituting 2-naphthyl-L-alanine for 2-naphthyl-D-alanine and 1-naphthyl-L-alanine, as well as changing the tranexamic acid for one, two, three, or four 4-aminomethylbenzoic acid building blocks. However, none of these modifications resulted in a radioligand with overall improved properties, with the authors suggesting that further studies are necessary [17].

**Figure 2:**
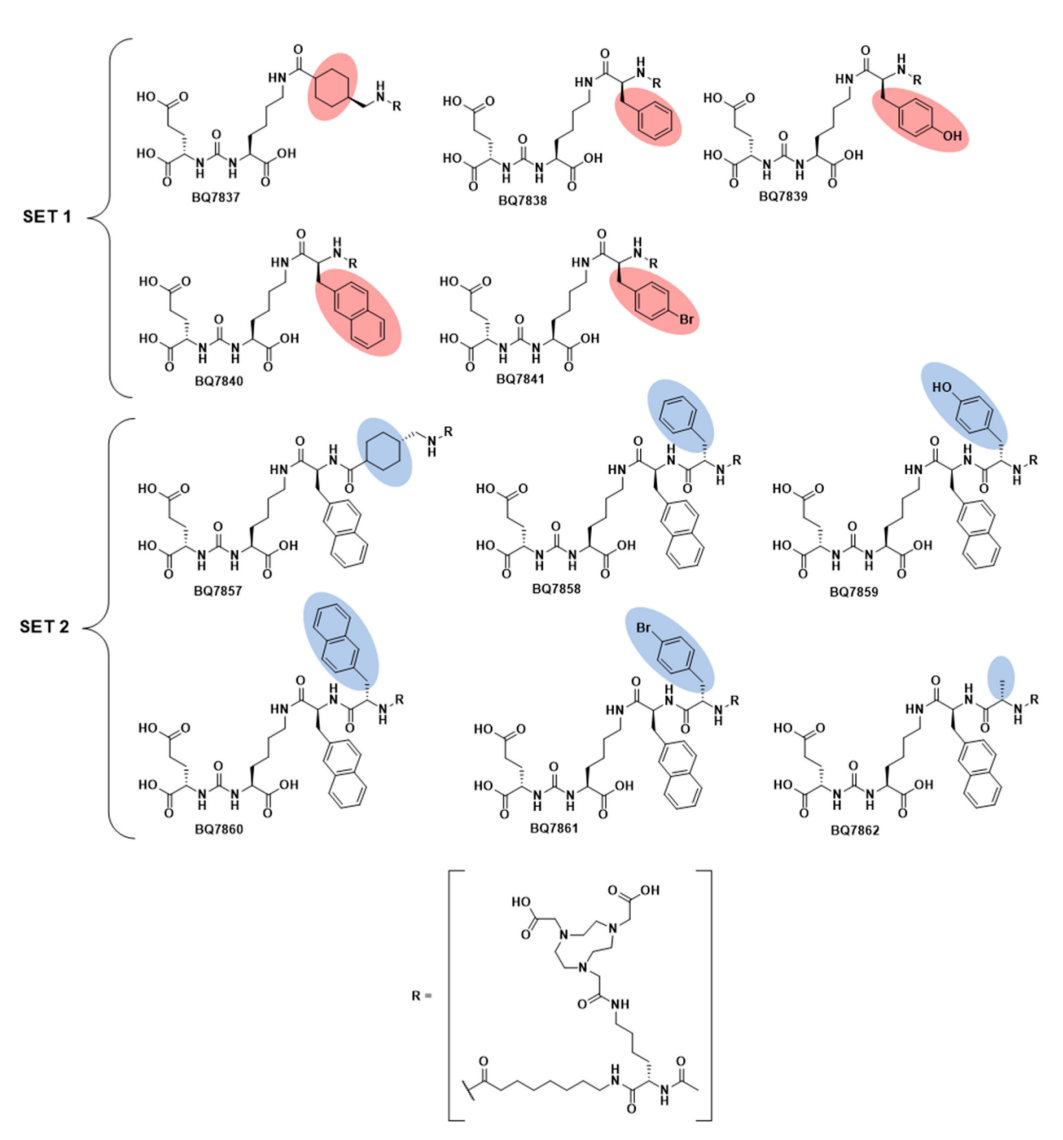
Chemical structure of compound BQ7837-BQ7841 (SET 1) and BQ7857-BQ7862 (SET 2).

To further investigate the relationship between the molecular structure and targeting properties, we herein present the development and evaluation of 11 novel PSMA-binding radioligands with structural modifications of the linker (Figure 2). The linker structure of PSMA-617 was used as a starting point for the linker design. The study was carried out by first examining the structural importance of the naphthylic linker (highlighted in red, Figure 1) targeting the S1 hydrophobic pocket, replacing it with other substituents and investigating how that influenced the targeting properties. Thereafter, cyclohexyl functionality in tranexamic acid (highlighted in blue, Figure 1) was investigated using computational modelling as a structural guidance. Modification at this position was done to investigate if this could lead to improved affinity and pharmacokinetic properties.

## 2. Materials and Methods

Detailed information regarding the methods, instruments, solvents, chemicals, and cell maintenance used can be found in Appendix A.

### 2.1. Computational Modelling

All calculations were performed within the Schrodinger Small-Molecule Drug Discovery Suite 2019-3 using the OPLS3 force field. The crystal structure of PSMA (human glutamate carboxypeptidase II (GCPII)) was downloaded from the Protein Data Bank (PDB entry: 5O5U) and thereafter prepared using the Protein Preparation Wizard, implemented in Maestro using default settings. This included the addition of hydrogens and hydrogen-bond network optimization. All water molecules were removed. To evaluate if the substituted analogue could interact with subpocket 2 (amino acids Phe546, Trp541, and Arg43), its structure was built into the protein using the protein-bound PSMA-1027 as a template. The analogue was truncated, after the substituent was added, to an acetyl group. The resulting complex was energy-minimized, where the ligand and amino acid side-chains within 5 Å from the added substituent were free to move. Minimal movement in the side chains was observed. The resulting complex was refined using 1000 steps of MCMM conformational sampling in a GB/SA continuum solvation model for water. During the conformational sampling, the newly attached substituent was sampled, whereas the rest of the ligand and the protein were kept frozen. This was done in the same way for all analogues in SET 2 (BQ7857-62).

### 2.2. Synthesis

#### 2.2.1. Di-tert-butyl 2-isocyanatopentanedioate (**1**)

**Scheme 1:**
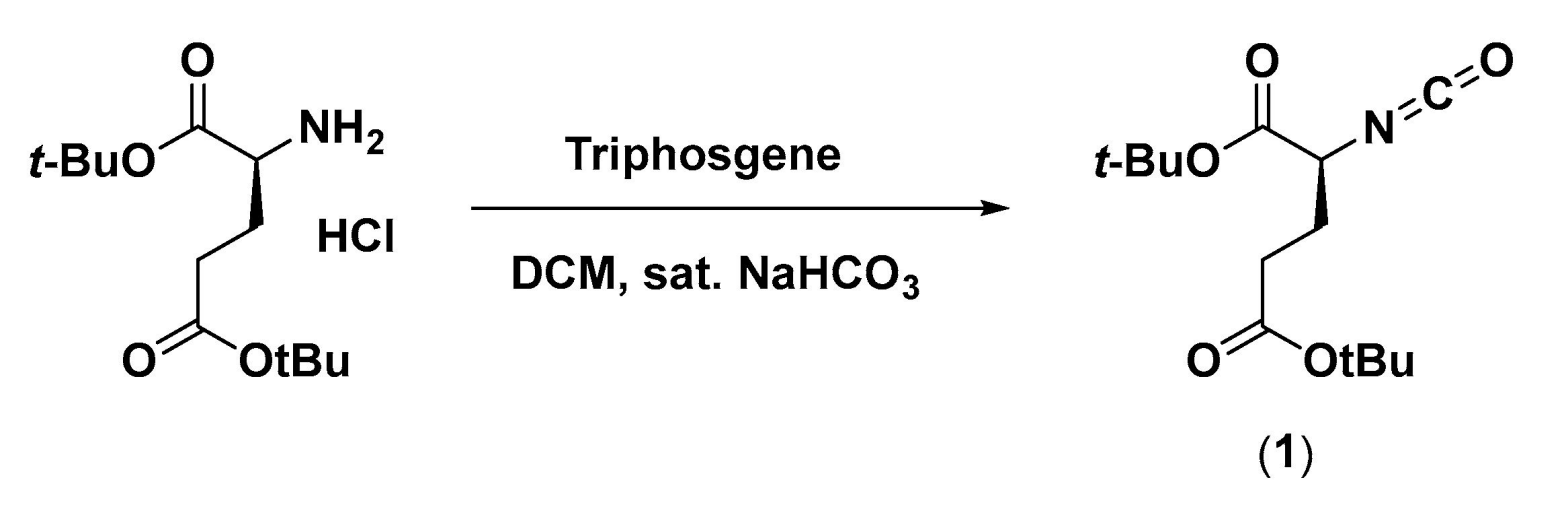
Synthetic scheme of di-tert-butyl 2-isocyanatopentanedioate (**1**).

Synthesis of **1** was done according to Scheme 1. Triphosgene (237 mg, 0.80 mmol) was dissolved in 10 mL dichloromethane (DCM). An amount of 10 mL sat. NaHCO_3_ was added and the mixture was stirred vigorously. L-Glu(*t*Bu)-O*t*Bu (591 mg, 2.0 mmol) was added. After 20 min, the reaction was checked with thin-layer chromatography (TLC), which indicated product formation. The organic phase (DCM) was separated from the aqueous phase and thereafter washed with 2 M KHSO_4_ × 2, dried with MgSO4, filtered, and evaporated. The product was obtained as a yellow oil (420 mg, 1.47 mmol).

#### 2.2.2. Di-tert-butyl (((S)-6-amino-1-methoxy-1-oxohexan-2-yl)carbamoyl)-L-glutamate (**2**)

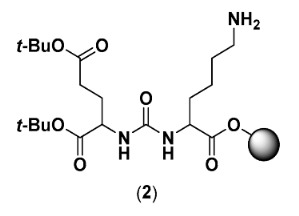



Synthesis of **2** was carried out by standard Fmoc solid-phase peptide synthesis (SPPS) using 2-chlorotrithyl resin (2-CTC) with a loading of 1.6 mmol/g as a solid support. To activate the resin (156 mg, 0.55 mmol), thionyl chloride (3 mL) and pyridine (2 mL) were added to the resin and the suspension was left for 16 h. The resin was filtered off and washed with DCM extensively. To couple the first amino acid, 2 equiv. of Fmoc-Lys(alloc)-OH (498 mg, 1.1 mmol) and 4 equiv. of diisopropylethylamine (DIPEA) (383 µL, 2.2 mmol) were dissolved in DCM, added to the resin, and left to react overnight. Thereafter, the resin was washed with DCM, Fmoc was removed using 20% piperidine in dimethylformamide (DMF) (3 × 10 min), and the resin was washed again with DMF followed by DCM. 2.7 equiv. of freshly prepared **1** (420 mg, 1.47 mmol) and 7 equiv. of DIPEA (671 µL, 3.85 mmol) were dissolved in DCM and added to the resin to form the urea. After 4 h, the resin was washed DCM. The alloc-protected lysine side chain was deprotected by the addition of PhSiH_3_ (20 Equiv.) and Pd(PPh_3_)_4_ (0.3 Equiv.) in DCM for 3 h. The resin was washed with DCM and DMF, and a small portion of the crude was cleaved off the resin and analyzed by analytical liquid-chromatography mass spectrometry (LC-MS) to confirm the correct mass of the product. Compound **2** was further used in the synthesis of BQ7837-BQ7841 and BQ7857-BQ7862.

#### 2.2.3. General Procedure for BQ7837-41 (SET 1)

All coupling reactions were performed in DMF using Oxyma Pure, N,N’-diisopropylcarbodiimide (DIC), and DIPEA. After each coupling, the resin was washed with DMF, Fmoc was removed using 20% piperidine in DMF, and the resin was washed again with DMF. After each coupling, a small portion was cleaved off the resin and the crude was analyzed by analytical LC-MS to confirm the correct mass of the wanted product.

BQ7837-41 were synthesised based on the general structure of Glu-urea-Lys-**X**-8Aoc-Lys(NOTA)-acetamide, where **X** was the position of substitution. Compound **2** (50 µmol) was swelled in DMF for 30 min. In a separate vial, **X** (0.2 mmol), Oxyma Pure (28 mg, 0.2 mmol), DIC (31 uL, 0.2 mmol), and DIPEA (61 µL, 0.35 mmol) were dissolved in DMF and thereafter added to **2**. The reaction was left stirring for 3 h. In the same way, Fmoc-8-Aoc-OH and Fmos-Lys(alloc)-OH were coupled, one after another, to the resin. After Fmoc-deprotection of lysine, the free amine at the N-terminal was capped by the addition of 2 mL acetic anhydride and pyridine (3:2). After 30 min, the capping solution was removed, and the resin was washed with DCM. The Alloc-protected lysine side chain was deprotected using PhSiH_3_ (20 eq.) and Pd(PPh_3_)_4_ (0.3 eq) in DCM for 3 h. As a final coupling, NOTA-bis(*t*Bu)ester (42 mg, 0.1 mmol), Oxyma Pure (14 mg, 0.1 mmol), DIC (15 uL, 0.1 mmol), and DIPEA (30 µL, 0.175 mmol) were dissolved in DMF, added to the resin, and left to react overnight. The product was cleaved from the resin using 5% water in trifluoroacetic acid (TFA). Thioanisole (120 µL) and triisopropylsilane (60 µL) were used as carbocation scavengers in the cleavage due to the simultaneous cleavage of acid-labile protecting groups. The crude product was precipitated in cold diethyl ether, purified by reversed-phase high-performance liquid chromatography (RP-HPLC), and freeze-dried. Analytical LC-MS was used to confirm the identity and purity of the product.

##### BQ7837

Compound BQ7837 was synthesised according to the general procedure described above, where **X** = Fmoc-tranexamic acid (76 mg, 0.2 mmol). Yield 6.1 mg. Calculated [M + H]^+^ and [M + 2H]^2+^: 1055.59 and 528.30. Observed: [M + H]^+^ and [M + 2H]^2+^: 1055.4 and 528.1 (Figure S2).

##### BQ7838

Compound BQ7838 was synthesised according to the general procedure described above, where **X** = Fmoc-Phe-OH (77 mg, 0.2 mmol). Yield 5.6 mg. Calculated [M+H]^+^ and [M + 2H]^2+^: 1063.56 and 532.28. Observed: [M + H]^+^ and [M + 2H]^2+^: 1063.3 and 532.2 (Figure S3).

##### BQ7839

Compound BQ7839 was synthesised according to the general procedure described above, where **X** = Fmoc-Tyr(tBu)-OH (92 mg, 0.2 mmol). Yield 6.1 mg. Calculated [M + H]^+^ and [M + 2H]^2+^: 1079.55 and 540.28 Observed: [M + H]^+^ and [M + 2H]^2+^: 1079.4 and 540.1 (Figure S4).

##### BQ7840

Compound BQ7840 was synthesised according to the general procedure described above, where **X** = Fmoc-2-Nal-OH (87 mg, 0.2 mmol). Yield 4.0 mg. Calculated [M + H]^+^ and [M + 2H]^2+^: 1113.48 and 557.24. Observed: [M + H]^+^ and [M + 2H]^2+^: 1113.3 and 557.1 (Figure S5).

##### BQ7841

Compound BQ7841 was synthesised according to the general procedure described above, where **X** = Fmoc-Phe(4-Br)-OH (93 mg, 0.2 mmol). Yield 5.4 mg. Calculated [M + H]^+^ and [M + 2H]^2+^: 1141.47/1143.47 and 571.24/572.24. Observed: [M + H]^+^ and [M + 2H]^2+^: 1141.3/1143.3 and 571.0/572.0 (bromine isotope pattern was observed) (Figure S6).

#### 2.2.4. Di-tert-butyl (((S)-6-((S)-2-amino-3-(naphthalen-2-yl)propanamido)-1-methoxy-1-oxohexan-2-yl)carbamoyl)-L-glutamate (**3**)

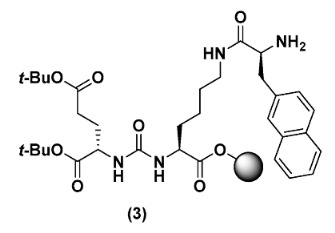



To synthesise **3**, compound **2** (0.3 mmol) was swelled in DMF for 30 min. In a separate vial, Fmoc-2-Nal-OH (525 mg, 1.2 mmol), Oxyma Pure (171 mg, 1.2 mmol), DIC (188 µL, 1.2 mmol), and DIPEA (366 µL, 2.1 mmol) were dissolved in DMF and thereafter added to the resin. The reaction was left stirring for 3 h. After washing with DMF, Fmoc was deprotected using 20% piperidine in DMF (3 × 10 min), and the resin was washed again with DMF followed by DCM. The resin was portioned into six separate reaction vessels with 50 µmol in each for the further synthesis of BQ7857-BQ7862.

#### 2.2.5. General Procedure for BQ7857-62 (SET 2)

The coupling reactions, resin wash, Fmoc-deprotection, monitorisation of the reactions, cleavage, purification, and analysis were performed in the same way as described for BQ7837-BQ7841.

BQ7857-62 was synthesised based on the general structure Glu-urea-Lys-2-naphthyl-L-alanine-**Y**-8Aoc-Lys(NOTA)-acetamide, where **Y** was the position of the substitution. Compound **3** (50 µmol) was swelled in DMF for 30 min. In a separate vial, **Y** (0.2 mmol), Oxyma Pure (28 mg, 0.2 mmol), DIC (31 uL, 0.2 mmol), and DIPEA (61 µL, 0.35 mmol) were dissolved in DMF and thereafter added to **3**. After 3 h, the resin was washed with DMF and Fmoc was deprotected. In the same way, Fmoc-8-Aoc-OH and Fmos-Lys(alloc)-OH were coupled after each other to the resin. After the Fmoc-deprotection of lysine, capping of the free amine at the N-terminal was done by the addition of 2 mL acetic anhydride and pyridine (3:2). After 30 min, the capping solution was removed, and the resin was washed with DCM. The Alloc-protected lysine side chain was deprotected using PhSiH_3_ (20 eq.) and Pd(PPh_3_)_4_ (0.3 eq) in DCM for 3 h. As a final coupling, NOTA-bis(*t*Bu)ester (42 mg, 0.1 mmol), Oxyma Pure (14 mg, 0.1 mmol), DIC (15 uL, 0.1 mmol), and DIPEA (30 µL, 0.175 mmol) were dissolved in DMF, added to the resin, and left to react overnight. The product was cleaved from the resin, precipitated in cold diethyl ether, and purified using RP-HPLC. Analytical LC-MS was used to confirm the identity and purity of the product.

##### BQ7857

Compound BQ7857 was synthesised according to the general procedure described above, where **Y** = Fmoc-tranexamic acid (76 mg, 0.2 mmol). Yield: 4.5 mg. Calculated [M + H]^+^ and [M+2H]^2+^: 1252.7 and 626.8. Observed: [M + H]^+^ and [M + 2H]^2+^: 1252.8 and 626.7 (Figure S7).

##### BQ7858

Compound BQ7858 was synthesised according to the general procedure described above, where **Y =** Fmoc-Phe-OH (77 mg, 0.2 mmol). Yield 3.0 mg. Calculated [M + H]^+^ and [M + 2H]^2+^: 1260.6 and 630.8. Observed: [M + H]^+^ and [M + 2H]^2+^: 1260.8 and 639.8 (Figure S8).

##### BQ7859

Compound BQ7859 was synthesised according to the general procedure described above, where **Y** = Fmoc-Tyr(tBu)-OH (92 mg, 0.2 mmol). Yield 4.8 mg. Calculated [M + H]^+^ and [M + 2H]^2+^: 1276.6 and 638.8. Observed: [M + H]^+^ and [M + 2H]^2+^: 1276.8 and 638.8 (Figure S9).

##### BQ7860

Compound BQ7860 was synthesised according to the general procedure described above, where **Y** = Fmoc-2-Nal-OH (87 mg, 0.2 mmol). Yield 3.8 mg. Calculated [M + H]^+^ and [M + 2H]^2+^: 1310.7 and 655.8. Observed: [M + H]^+^ and [M + 2H]^2+^: 1310.9 and 655.7 (Figure S10).

##### BQ7861

Compound BQ7861 was synthesised according to the general procedure described above, where **Y** = Fmoc-Phe(4-Br)-OH (93 mg, 0.2 mmol). Yield 2.9 mg. Calculated [M + H]^+^ and [M + 2H]^2+^: 1340.4 and 670.7. Observed: [M + H]^+^ and [M + 2H]^2+^: 1340.8 and 670.6 (bromine isotope pattern was observed) (Figure S11).

##### BQ7862

Compound BQ7862 was synthesised according to the general procedure described above, where **Y** = Fmoc-Ala-OH (62 mg, 0.2 mmol). Yield 5.4 mg. Calculated [M + H]^+^ and [M + 2H]^2+^: 1184.6 and 592.8. Observed: [M + H]^+^ and [M + 2H]^2+^: 1184.7 and 592.9 (Figure S12).

### 2.3. Radiochemistry

The PSMA-binding compounds BQ7837-41 and BQ7857-62 were dissolved in 0–30% EtOH in MQ water (1 nmol/μL). Ammonium acetate buffer (65 μL, 0.2 M, pH 5.5) was added to a LoBind Eppendorf tube followed by the addition of the PSMA-binding compound of interest (5 μL, 5 nmol) and [^111^In]InCl_3_ (30 μL, 25 MBq). The reaction mixture was left for 30 min at 85 °C. The crude reaction mixture was analysed by instant thin-layer chromatography (ITLC), eluted with 0.2 M citric acid and reversed-phase radio-HPLC. To evaluate the stability, the radiolabelled compound was added to a solution of EDTA (1000-fold molar excess) and PBS and left at room temperature. After 1, 3, and 24 h incubations, the solutions were analysed by ITLC (eluent 0.2 M citric acid) to determine the labelling stability. The radio-HPLC spectra of compounds radiolabelled with indium-111 can be found in the supplementary materials.

### 2.4. Octanol/Water

The LogD was determined experimentally based on the procedure described earlier [25]. Briefly, the radiolabelled compound of interest was added to a LoBind Eppendorf tube containing Milli-Q water (500 μL) and *n*-octanol (500 μL) and the mixture was centrifuged and vortexed. Thereafter, three fractions from each phase were collected and the activity was measured using a gamma counter. The LogD was calculated by dividing the average activity measured in octanol by the average activity measured in water.

### 2.5. In Vitro Characterisation

#### 2.5.1. Specific Binding to PSMA

Approximately 3 × 10^5^ cells/well were seeded in 12-well plates 24 h before the experiment. To saturate the target, two sets of cells were pre-treated with either non-labelled PSMA-11 (500 nM/well) or a non-labelled variant of the PSMA-binding compound of interest (500 nM/well), while the third set was treated with media. After 15 min incubation at room temperature, 1 nM/well of the radiolabelled compound was added to all wells and cells were incubated at 37 °C. After 1 h, media were aspirated, and cells were treated with trypsin. The activity of the detached cells was measured using a gamma counter.

#### 2.5.2. Cellular Retention

To evaluate the in vitro cellular retention of each compound, 1 nM of the radiolabelled compound was added to each well and cells were incubated at 4 °C. After 1 h, the media were aspirated and fresh complete media were added followed by incubation at 37 °C (determined to time point 0 h). At pre-determined time points (1, 4, and 24 h), the media were aspirated, the cells detached by the addition of trypsin, and activity was measured using a gamma counter. At 4 and 24 h time points, the cell-associated activity was measured. This was done according to the procedure described in the literature [26]. Briefly, the cells were first treated with acid wash (0.2 M glycine buffer, 0.15 M NaCl, 4 M Urea, pH 2) to collect the membrane-bound fraction, followed by the treatment of base wash (1 M NaOH) to collect the internalized fraction.

#### 2.5.3. Real-Time Measurement of Binding Kinetics

The binding kinetics (K_a_ and K_d_) were measured in real-time using a yellow Ligand Tracer instrument. Approximately three million PC3-pip cells were seeded in a designated area of a Petri dish with increased surface binding. The PSMA-binding ligand of interest radiolabelled with indium-111 was added in pre-determined concentrations (1, 3, and 10 nM) to measure the association rate (K_a_). The concentration of the radiolabelled compound was increased stepwise when the binding curve at the previous concentration had reached an equilibrium. Thereafter, the radioactive solution was replaced with cell culture media to measure the dissociation rate (K_d_). K_a_ and K_d_ were computed using a 1:1 kinetic binding model in TraceDrawer software. K_D_ was calculated by dividing K_d_ with K_a_.

### 2.6. In Vivo Characterisation

The in vivo studies were conducted according to the guidelines of the Declaration of Helsinki and approved by the Ethics Committee for Animal Research in Uppsala, Sweden Approval number: 5.8.18-00473/2021. Approved on 26 February 2021.

#### 2.6.1. Biodistribution

The biodistribution of [^111^In]In-BQ7857-61 was studied at 1 h pi. Mice were intravenously injected with 40 pmol (30 kBq) of the radiolabelled PSMA-binding ligand of interest dissolved in a total volume of 100 μL 1% Bovine Serum Albumin (BSA) in PBS. At 1 h pi., mice were euthanised after pre-injection of Ketalar–Rompun solution (10 mg/mL Ketalar and 1 mg/mL Rompun; 20 μL solution/g body weight) and the organs of interest were collected and weighed. Thereafter, the uptake activity in organs was measured using a gamma counter and calculated as the percentage of injected dose per gram (%ID/g) for blood, salivary gland, lung, liver, spleen, large intestine, kidney, tumour, muscle, and bone tissue, and as %ID for the GI and the remaining carcass. For [^111^In]In-BQ7857 and [^111^In]In-BQ7859, the biodistribution was studied at 24 h following the same procedure as described for 1 h time point.

#### 2.6.2. Specific Binding to PSMA

Specific binding to PSMA was studied in vivo for [^111^In]In-BQ7859 by the intravenous injection of [^111^In]In-BQ7859 (40 pmol, 30 kBq) in a total volume of 100 μL 1% BSA in PBS. Mice in the blocked group were co-injected with 5 nmol non-labelled PSMA-11 to block PSMA. Animals were euthanised, organs were collected, and uptake activity measured according to the protocol described above.

### 2.7. Statistical Analysis

Statistical analysis was performed using GraphPad Prism 8.0 for Windows (GraphPad Software, San Diego, CA, USA). An unpaired, two-tailed *t*-test was assessed to determine statistical significance between two groups and a one-way ANOVA with the Bonferroni test corrected for multiple comparisons was assessed to determine statistical significance between more than two groups.

## 3. Results and Discussion

### 3.1. Evaluation of BQ7837-41 (SET 1)

The development of PSMA-617 was a result of linker modification of urea-based PSMA-binding inhibitors, and has since shown promising results as a theranostic radiotracer due to its favourable targeting properties [23]. The reason for the improved characteristics of PSMA-617 is predominantly due to its naphthylic linker. Thus, the first objective of this study was to examine the modifications at the naphthylic position of the linker. Based on the structure of PSMA-617, five analogues (Figure 2) with different linkers were designed and synthesised with the general structure of Glu-urea-Lys-**X**-8Aoc-Lys(NOTA)-acetamide, where **X** was the position of substitution. To cover a range of size, hydrophobicity, and the possibility to form additional interactions with the amino acids in the S1 hydrophobic pocket, **X** was substituted for tranexamic acid (BQ7837), L-phenylalanine (BQ7838), L-tyrosine (BQ7839), 2-naphthyl-L-alanine (BQ7840), and L-(4-bromo)phenylalanine (BQ7841). BQ7840 was considered as a reference for comparison since its linker contains the same naphthylic structure as in PSMA-617. To distance the radiometal chelator from the modified linker structure, -8Aoc- was incorporated into the molecules. This set of analogues is further denoted as SET 1. After synthesis, BQ7837-41 were successfully radiolabelled with indium-111 with an average radiochemical yield of 98.9 ± 0.3% (Table 1) in molar activities up to 30 MBq/nmol. After EDTA challenge, the average detected release of free indium-111 detected was 2.2 ± 0.9% (Table 1), demonstrating stable [^111^In][In-NOTA] complexes for all five compounds.

**Figure 3:**
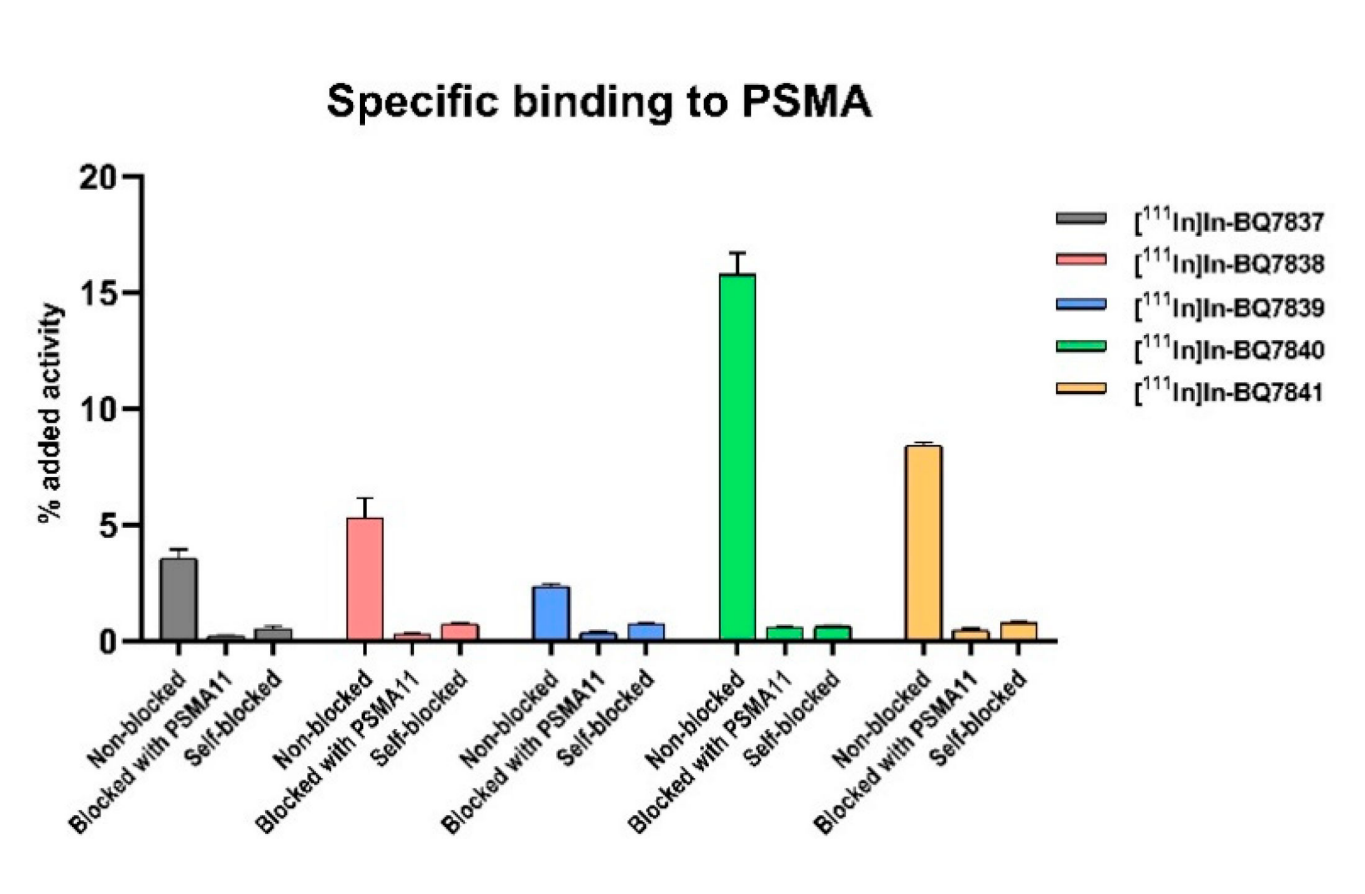
In vitro binding specificity of [^111^In]In-BQ7837-41 (SET 1) in PSMA-expressing PC3-pip cells. To block PSMA, 500 nM/well of non-labelled PSMA-11 or the non-labelled variant of the PSMA-binding ligand of interest were used. For all analogues, the same concentration of radiolabelled PSMA-binding radioligand was added to all wells. Statistical analysis was done using an unpaired, two-tailed *t*-test.

To evaluate the specific binding to PSMA of [^111^In]In-BQ7837-41 in SET 1, an in vitro specificity study was performed using PSMA-expressing PC3-pip cells. For all five analogues, the uptake was significantly (*p* < 0.05) reduced in cells that were pre-treated with an excess of non-labelled PSMA-11 or a non-labelled variant of itself (self-blocked) when compared to non-treated cells, demonstrating the specific binding to the target (Figure 3). When comparing the activity uptake of the different analogues, a higher uptake of [^111^In]In-BQ7840 (~15%) could be seen when compared with the other four analogues (<8%). This indicated a higher affinity and more beneficial binding properties of [^111^In]In-BQ7840. To investigate the affinity to PSMA further, the binding kinetics were measured in a real-time experiment. As hypothesised based on the results of the specificity study, [^111^In]In-BQ7840 demonstrated a higher affinity to PSMA when compared with the other four analogues (Table 1). The binding affinity of [^111^In]In-BQ7840 was in the low nanomolar range and was consistent with the binding affinity of PSMA-617. This was a logical outcome, as both compounds contain 2-naphthyl-L-alanine at the same position in their linkers [23]. The K_D_-value for [^111^In]In-BQ7840 was more than 700-fold lower when compared with [^111^In]In-BQ7837-39 and was around 30-fold lower than for [^111^In]In-BQ7841. This suggested that the size of the chemical structure interacting with the S1 hydrophobic pocket is important. Compounds [^111^In]In-BQ7840 and [^111^In]In-BQ7841, containing 2-naphthyl-L-alanine and L-(4-bromo)phenylalanine, respectively, are the analogues containing the largest substituents in their linkers and those with significantly better K_D_-values (4.39 nM and 147 nM). When comparing the affinity of [^111^In]In-BQ7838 (K_D_ = 7520 nM) and [^111^In]In-BQ7841 (K_D_ = 147 nM), it also appears that the lipophilicity of the substituent matters. L-phenylalanine is less lipophilic in comparison to L-(4-bromo)phenylalanine due to the higher π-value of bromine relative to hydrogen [27]. The results from the in vitro experiments of [^111^In]In-BQ7837-41 strongly indicate that the naphthylic structure of 2-naphthyl-L-alanine probably finds a highly beneficial position within the S1 hydrophobic pocket, and is thus crucial. Therefore, we decided to retain 2-naphthyl-L-alanine at this position (X) when designing the linker structures for investigating the importance of the cyclohexyl functionality in the linker.

### 3.2. Evaluation of BQ7857-62 (SET 2)

**Figure 4:**
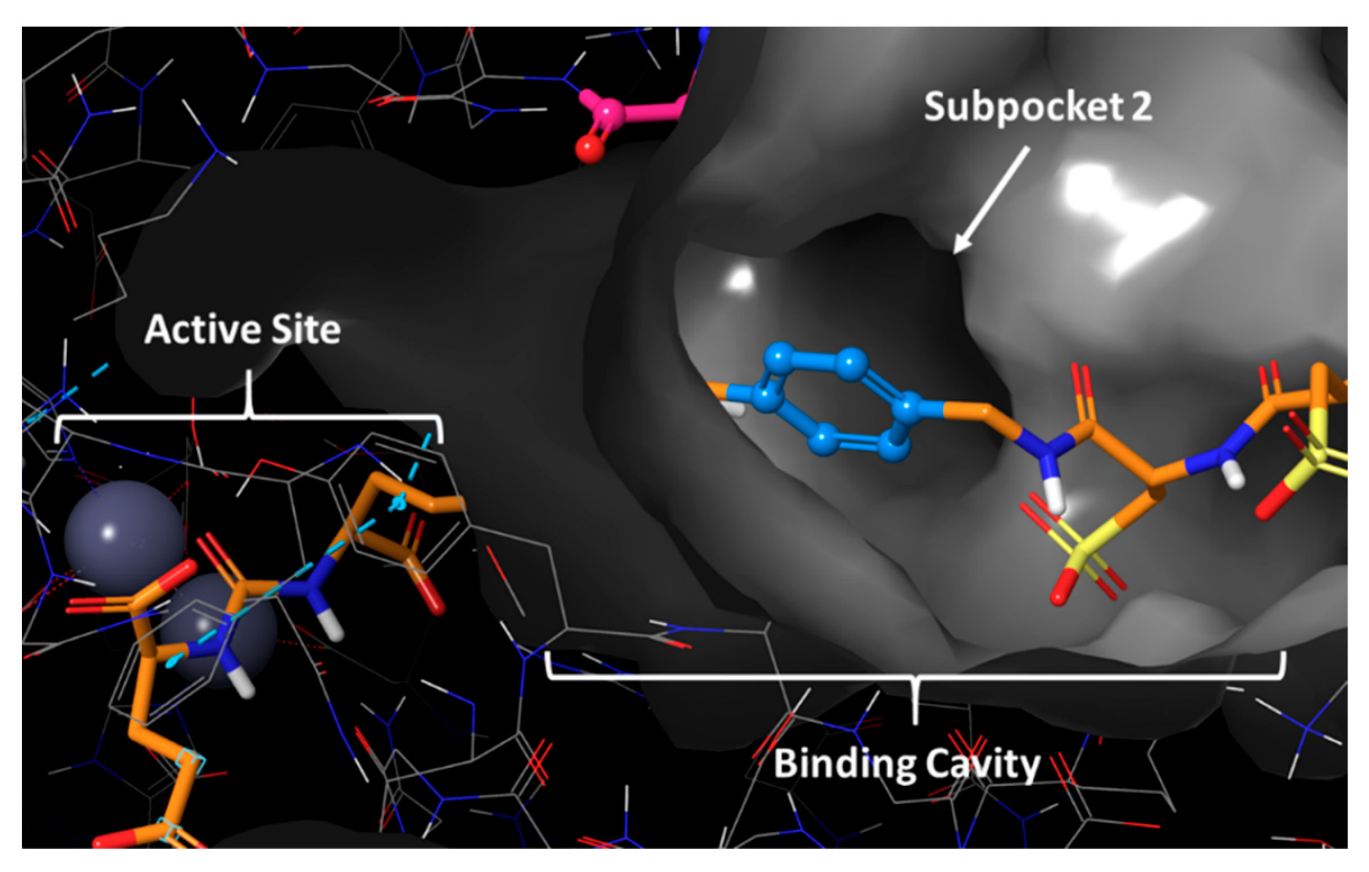
Crystal structure of PSMA and the co-crystallized ligand PSMA-1027 depicted in orange. PDB code 5O5U.

Structure-based design and computational modelling are techniques commonly used to minimize time and cost in drug development. X-ray crystal structures of known targets can provide valuable information on ligand binding, leading to the generation of ideas regarding how and at what position a compound can be modified. The second objective of this study was to investigate the structural importance of the cyclohexyl functionality in the linker of PSMA-617. To get a better understanding of the binding cavity and how tranexamic acid could be substituted, the crystal structure of PSMA was examined. Since there is no crystal structure of PSMA co-crystallised with PSMA-617 available in the Protein Data Bank (PDB), the X-ray crystal structure of PSMA co-crystallized with PSMA-1027 was used. The only relevant difference for this study in the linker structure of PSMA-1027 and PSMA-617 is the presence of 4-(aminomethyl)benzoic acid instead of tranexamic acid, i.e., a phenyl group in place of the cyclohexyl. After X-ray crystal structure analysis, a subpocket consisting of Phe546, Trp541, and Arg463 (further denoted as “subpocket 2”) was identified within the binding cavity that was located close to the cyclohexyl functionality of the linker (Figure 4). Based on this, we hypothesised the position of the cyclohexyl to be the optimal site for substitution, in order to enable interactions with amino acids in subpocket 2. It also appeared that subpocket 2 seemed to be smaller in size when compared to the S1 hydrophobic pocket.

**Figure 5:**
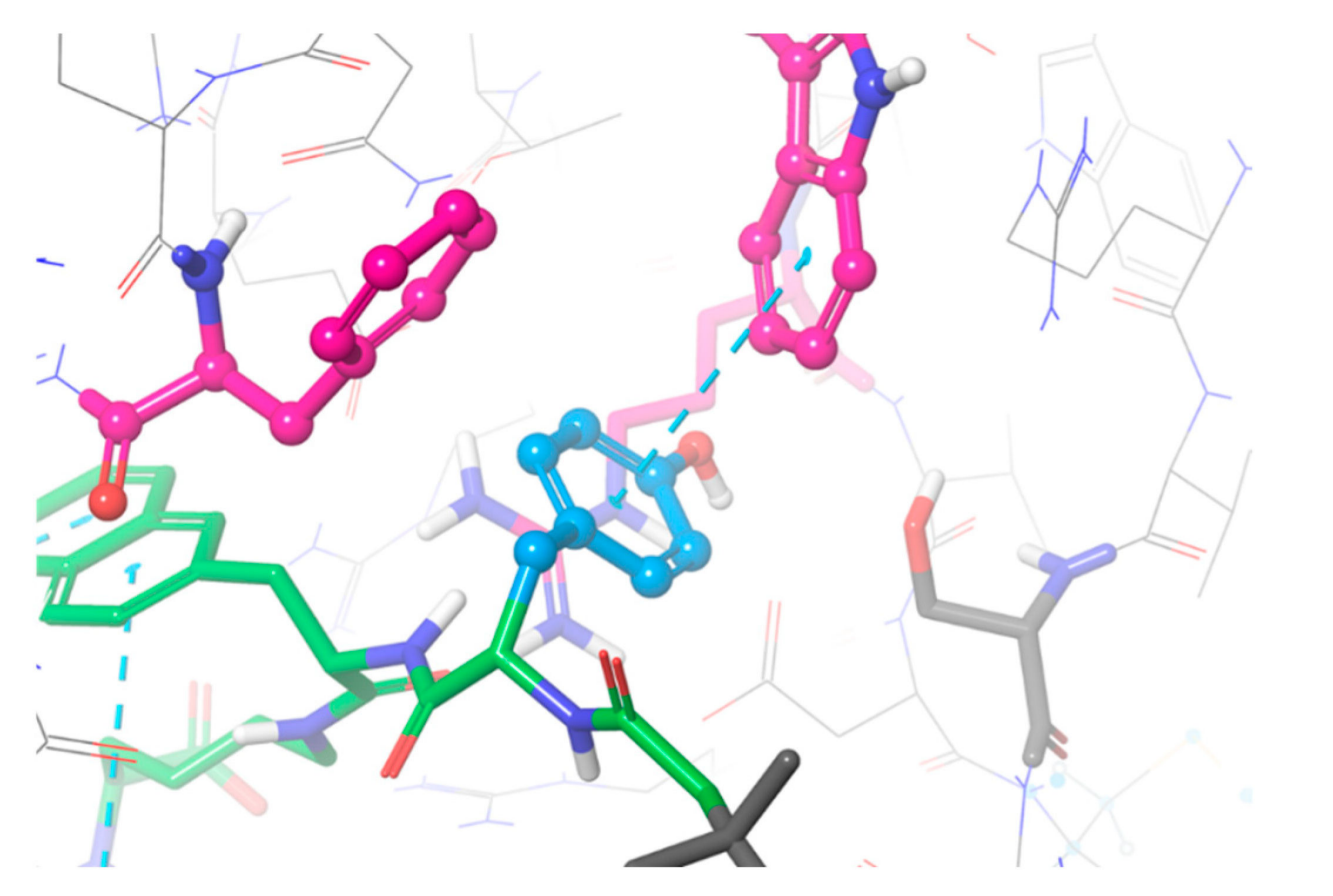
The suggested binding mode of compound BQ7859 depicted in green with the L-tyrosine substituent coloured in blue, interacting with amino acids Phe546, Trp541, and Arg463 within subpocket 2 depicted in pink.

With the general structure of Glu-urea-Lys-2-naphthyl-L-alanine-**Y**-8Aoc-Lys(NOTA)-acetamide, six novel PSMA-binding analogues (SET 2, Figure 2) were designed varying at position **Y**. Similar to SET 1, substituents differing in size, hydrophobicity, and their ability to form hydrogen and halogen bonds were chosen. As control and reference for the linker structure of PSMA-617, BQ7857 was designed, consisting of tranexamic acid in position **Y**. For the other analogues, **Y** was substituted for L-phenylalanine (BQ7858), L-tyrosine (BQ7859), 2-naphthyl-L-alanine (BQ7860), L-(4-bromo)phenylalanine (BQ7861), and L-alanine (BQ7862). After conformational sampling, it was concluded that these substituents could interact with the amino acids within subpocket 2. As seen in the suggested binding mode of BQ7859 in Figure 5, the amino acid sidechain of L-tyrosine occupies subpocket 2, making it possible to form additional interactions. The same binding mode was observed for the other substituents as well (Figure S1), which made us go further with the suggested substituents in position **Y**. The compound BQ7857-62 was synthesised using SPPS and radiolabelled with indium-111, with an average yield of 98.9 ± 0.6% (Table 2) in molar activities up to 30 MBq/nmol. The average release of free indium-111 after challenging with an excess of EDTA was 3.9 ± 2% (Table 2).

**Figure 6:**
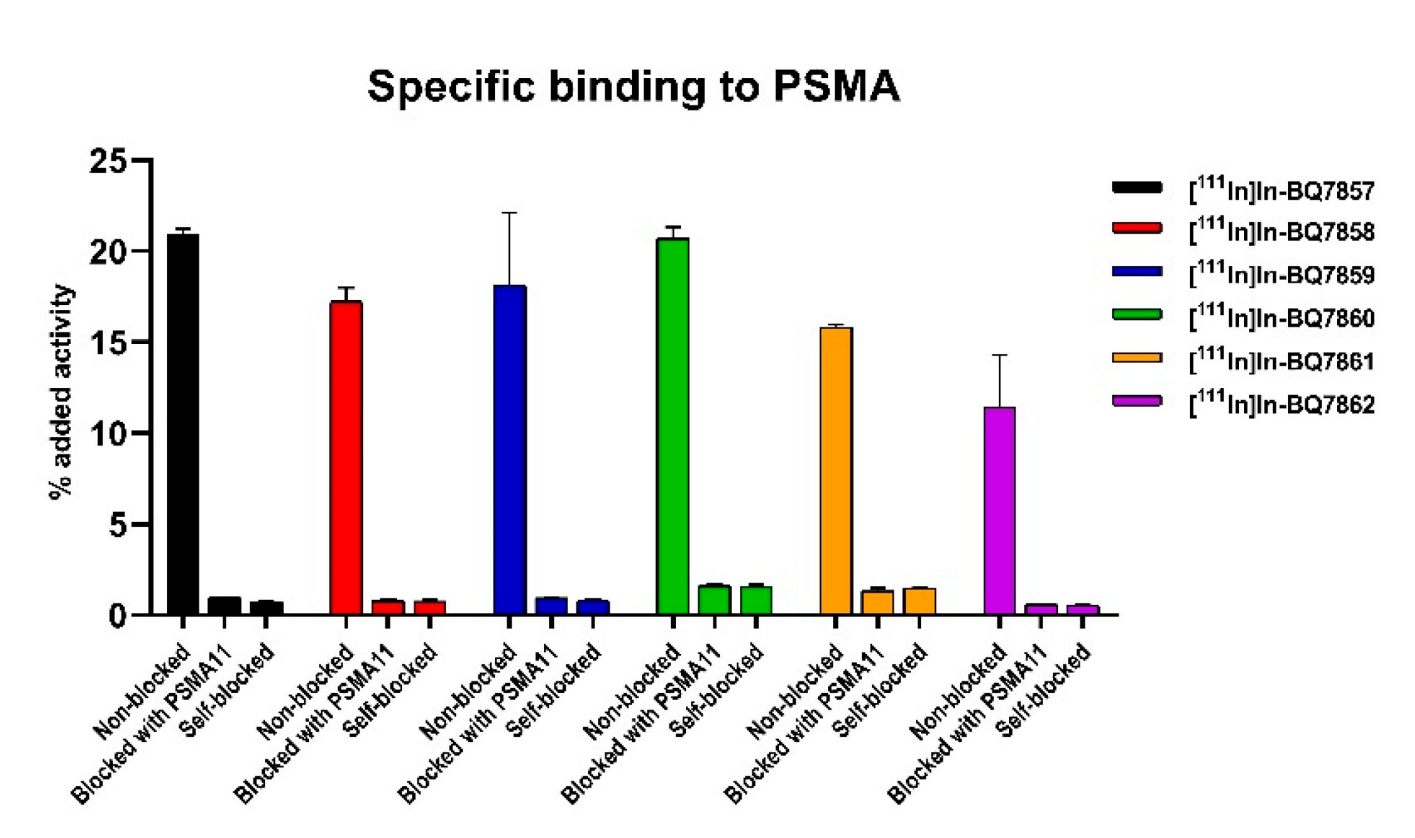
In vitro binding specificity of compounds [^111^In]In-BQ7857-62 (SET 2) in PSMA-expressing PC3-pip cells. To block PSMA, 500 nM/well of non-labelled PSMA-11 or a non-labelled variant of the compound of interest (self-blocked) were used. Statistical analysis was done using an unpaired, two-tailed *t*-test.

To evaluate the specific binding of compounds [^111^In]In-BQ7857-62 in SET 2, an in vitro specificity study was conducted in the same way as for SET 1. The uptake in cells pre-treated with an excess of non-labelled PSMA-11 or a non-labelled variant of itself (self-blocked) was significantly lower (*p* < 0.05) when compared with the non-treated cells (Figure 6). The activity uptake was over 10% of the added activity for all the compounds in SET 2. To investigate the affinity to PSMA, the binding kinetics were measured in real-time, resulting in affinities in the low nanomolar range for all six analogues (Table 2). Affinities for [^111^In]In-BQ7857-62 were in the same range as reported for [^68^Ga]Ga-PSMA-617 and [^68^Ga]Ga-PSMA-11 [23]. No large differences in the K_D_-value of the analogues were observed. This indicates that the substitution of tranexamic acid for L-phenylalanine, L-tyrosine, 2-naphthyl-L-alanine, L-(4-bromo)phenylalanine, or L-alanine does not seem to have a remarkable effect on affinity. However, the reference compound [^111^In]In-BQ7857, consisting of the same linker structure as PSMA-617, demonstrated the lowest K_D_-value (2.67 nM) and, thus, the highest affinity. The compounds with the highest K_D_-values were [^111^In]In-BQ7860 (K_D_ = 16.5 nM) and [^111^In]In-BQ7861 (K_D_ = 12.2 nM), containing 2-naphthyl-L-alanine and L-(4-bromo)phenylalanine, respectively. As observed from the X-ray analysis of PSMA, subpocket 2 seemed to be more restricted in size when compared to the S1 hydrophobic pocket. Together with the results from the affinity measurements, this suggests that large and hydrophobic substituents do not promote the binding, but instead slightly decrease the affinity.

**Figure 7:**
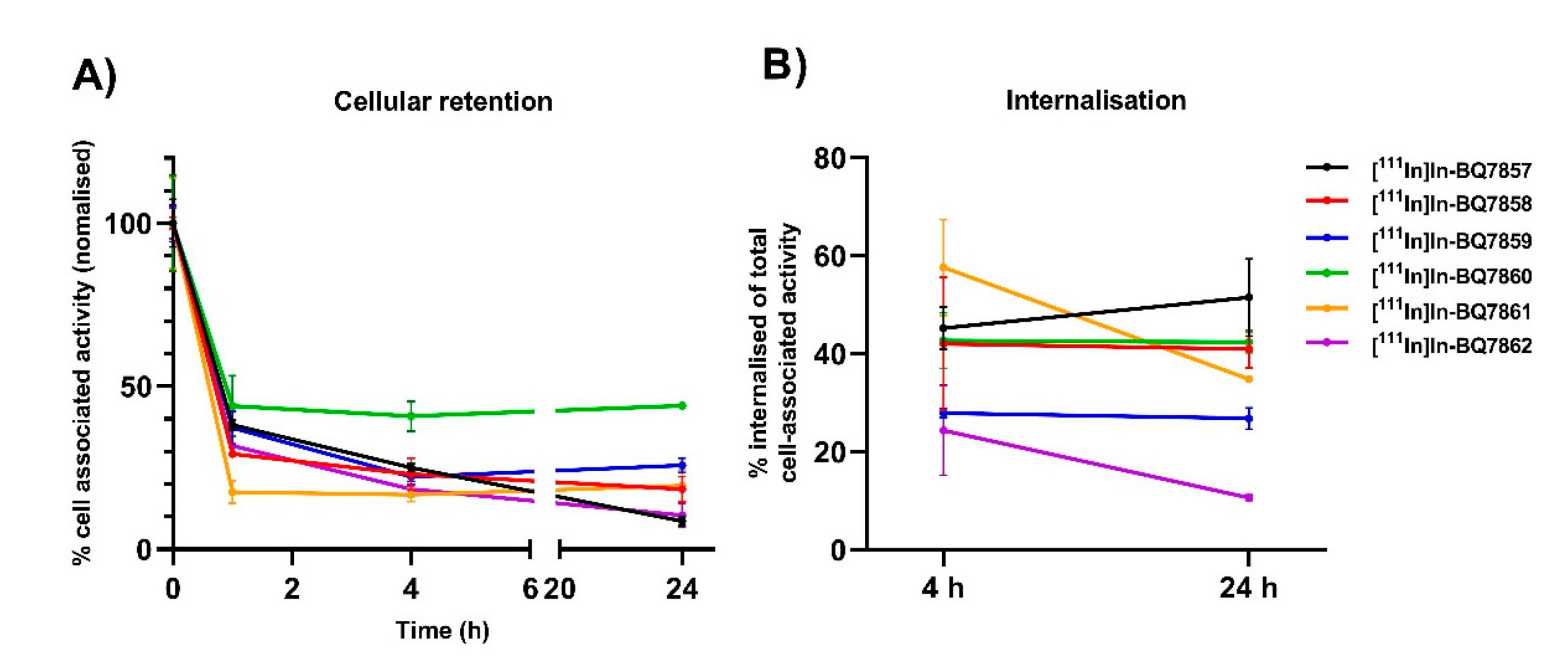
Cellular retention study using PSMA-expressing PC3-pip cells of [^111^In]In-BQ7857-62. (**A**) Total cell-associated activity at 1, 4, and 24 h. (**B**) Percentage of internalised activity based on the total cell-associated activity at 4 and 24 h time-points. Statistical analysis was done using a one-way ANOVA with the Bonferroni’s test, corrected for multiple comparisons.

An important feature for imaging agents is high target retention over time. To investigate this, a cellular retention study was conducted for SET 2. At 1, 4, and 24 h, the total cell-associated activity was measured, and at 4 and 24 h, the amount of internalised activity from the total cell-bound activity was determined. From the analysis of the total cell-associated activity, i.e., membrane-bound and internalised, [^111^In]In-BQ7860 demonstrated the highest value at all three timepoints (Figure 7A). This is potentially explained by its higher hydrophobicity (LogD = −1.42, Table 2), as more hydrophobic compounds tend to nonspecifically adhere to cells to a higher extent when compared to more hydrophilic compounds. The compound with the second-highest total cell-associated activity at 24 h was [^111^In]In-BQ7859, which had a significantly higher value when compared to the other four analogues. Turning to internalised activity, [^111^In]In-BQ7861 demonstrated the highest value at 4 h, which was significantly higher than for [^111^In]In-BQ7859 and [^111^In]In-BQ7861. No significant differences in internalised activity were observed for the other compounds at this time point. However, at 24 h, [^111^In]In-BQ7857, [^111^In]In-BQ7858, and [^111^In]In-BQ7860 demonstrated a higher amount of internalised activity when compared with the other three analogues (Figure 7B). The amount of internalised activity decreased for [^111^In]In-BQ7861 and [^111^In]In-BQ7862 from 4 to 24 h, while it was constant for [^111^In]In-BQ7857, [^111^In]In-BQ7858, [^111^In]In-BQ7859, and [^111^In]In-BQ7860. This might indicate the non-sufficient intracellular retention of [^111^In]In-BQ7861 and [^111^In]In-BQ7862. Based on the low total cell-associated activity, the low amount of internalised activity, and the significant reduction of internalised activity between 4 and 24 h, compound BQ7862 was excluded from further investigation in vivo.

**Figure 8:**
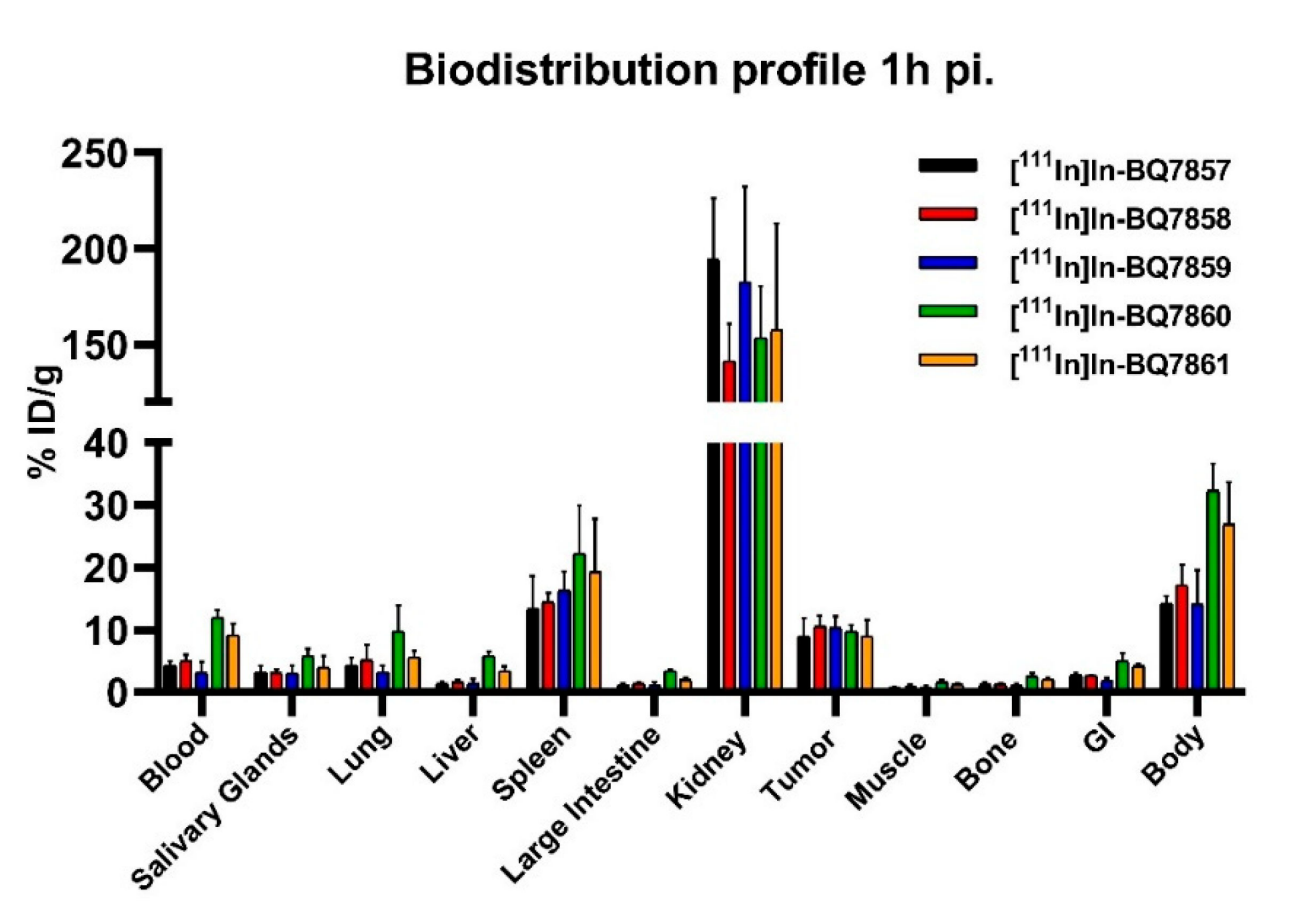
Biodistribution profile of [^111^In]In-BQ7857-61 in PSMA-expressing PC3-pip tumour-bearing mice 1 h post-injection (4 mice/group). Statistical analysis was done using a one-way ANOVA with the Bonferroni’s test corrected for multiple comparisons.

To investigate how the different linker structures of [^111^In]In-BQ7857-61 would affect the pharmacokinetic properties, an in vivo biodistribution study using PC3-pip tumour-bearing mice was conducted. The uptake of activity in organs of interest at 1 h post-injection is displayed in Figure 8 (numerical values are reported in Table S1). All the tested analogues demonstrated similar uptake in tumours (around 10% ID/g), the kidneys, spleen, and salivary glands. For [^111^In]In-BQ7860 and [^111^In]In-BQ7861, the two most lipophilic compounds based on the LogD values (Table 2), a significantly higher uptake in the blood, liver, and GI tract was observed. As high contrast is crucial for optimal imaging, [^111^In]In-BQ7860 and [^111^In]In-BQ7861 were excluded from further investigation in vivo. The comparison of [^111^In]In-BQ7858 and [^111^In]In-BQ7859 showed similar biodistribution profiles. However, when taking the results from both the in vitro and in vivo experiments into consideration, [^111^In]In-BQ7859 exhibits more beneficial properties as an imaging agent. This is due to its reduced uptake in blood and bone when compared with [^111^In]In-BQ7858, which would lead to higher tumour-to-organ (T/O) ratios. [^111^In]In-BQ7859 also demonstrated a higher amount of total cell-associated activity at 24 h (Figure 7A) and a slightly better K_D_-value (Table 2). Based on this, [^111^In]In-BQ7859 was chosen to be evaluated and compared with the reference compound [^111^In]In-BQ7857 in subsequent experiments.

**Figure 9:**
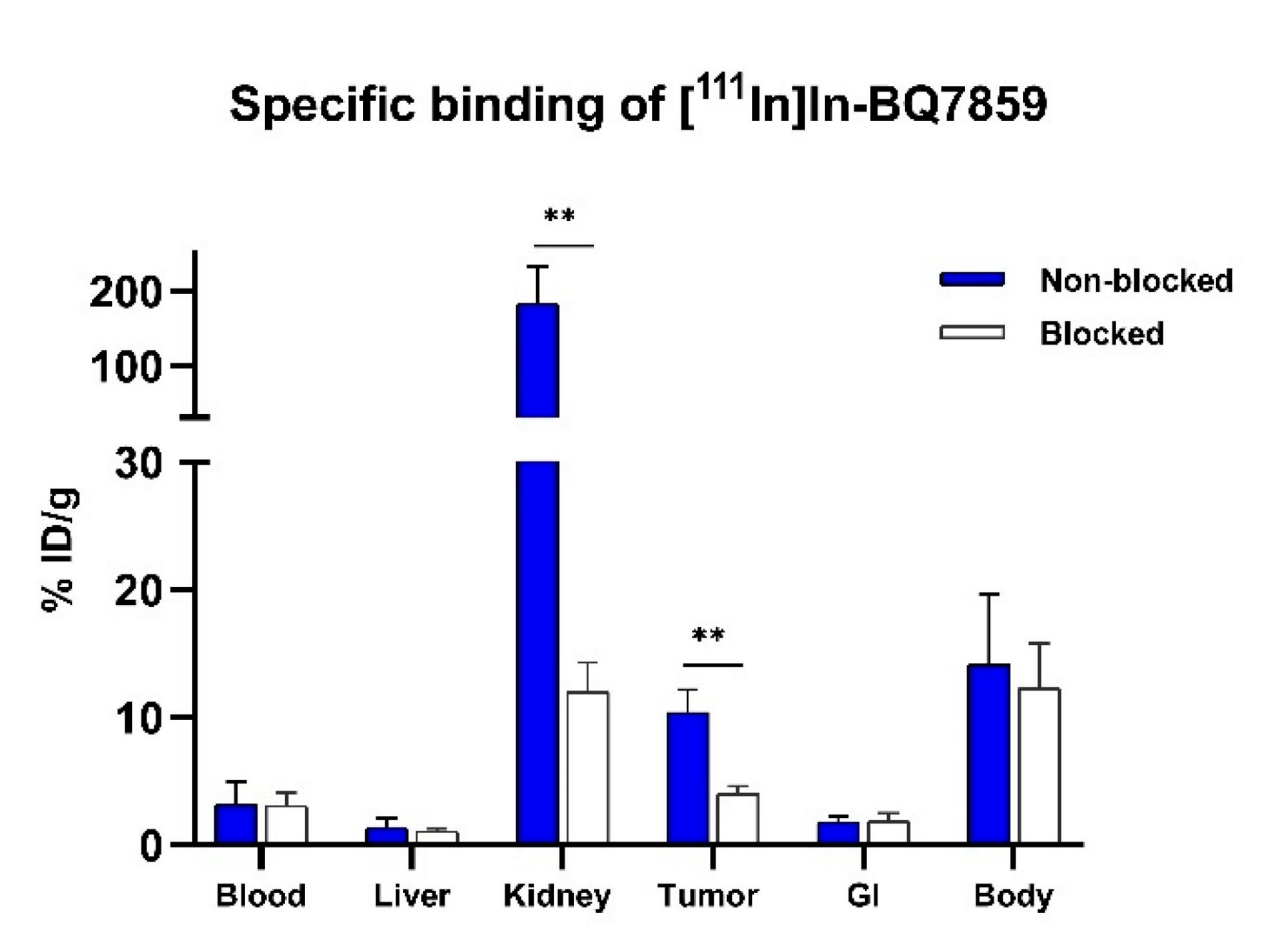
In vivo specificity of [^111^In]In-BQ7859 1 h post-injection in PSMA-expressing PC3-pip tumour-bearing mice. To block PSMA, 5 nmol non-labelled PSMA-11 was used. ** indicate *p*-value < 0.01. Statistical analysis was done using an unpaired, two-tailed *t*-test.

To confirm the specific binding of [^111^In]In-BQ7859 to PSMA, activity uptake in tumours and PSMA-expressing organs were compared between a non-blocked group injected with [^111^In]In-BQ7859 (40 pmol, 30 kBq) and a blocked group injected with [^111^In]In-BQ7859 (40 pmol, 30 kBq) + 5 nmol non-labelled PSMA-11. Results confirmed the earlier in vitro findings. A significantly lower uptake in both kidneys and tumours was seen in mice co-injected with an excess of cold blocking when compared with the non-blocked group (Figure 9). Since the specific binding of PSMA-617 to PSMA has already been proven [23], it was ethically motivated not to study the specific binding of [^111^In]In-BQ7857 in vivo, as the two compounds share both linkers and the same structure that binds to the active site.

**Figure 10:**
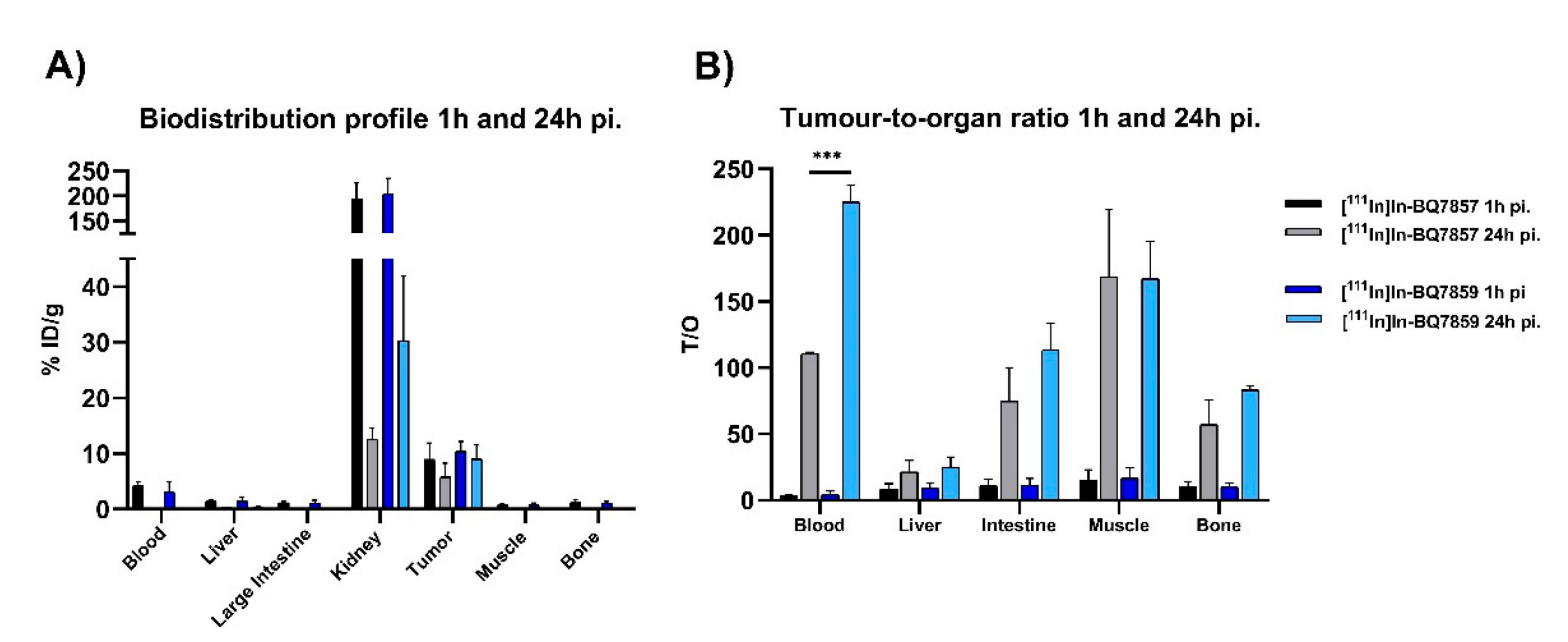
In vivo biodistribution of [^111^In]In-BQ7857 and [^111^In]In-BQ7859 in PSMA-expressing PC3-pip tumour-bearing mice at 1 and 24 h post-injection. (**A**) Uptake activity in specified organs. (**B**) Tumour-to-organ ratio (T/O). *** indicate *p*-value < 0.001. Statistical analysis was done using an unpaired, two-tailed *t*-test.

Results from the in vivo biodistribution study of [^111^In]In-BQ7857 and [^111^In]In-BQ7859 at 24 h pi. show a significantly higher activity uptake in the kidneys and tumours, when compared with their uptake in the blood, liver, large intestine, muscle, and bone (Figure 10A) (numerical values are reported in Table S2). This was expected, as both kidneys and the tumour express PSMA. The uptake in the kidneys is, however, significantly higher for [^111^In]In-BQ7859 when compared with [^111^In]In-BQ7857. This could be due to its more hydrophilic structure or stronger binding to PSMA. Since the difference in LogD value between the two analogues is minimal (−2.97 for [^111^In]In-BQ7857 and −3.03 for [^111^In]In-BQ7859), the stronger binding is the more likely reason. The same observations have been seen in previous studies where a higher affinity to PSMA led to an increased uptake activity in the kidneys [25,28]. The in vitro experiments showed that the binding affinity was within the same low nanomolar range for both compounds while the total cell-associated activity was significantly higher for [^111^In]In-BQ7859 (Figure 7A). Even though [^111^In]In-BQ7857 tends to have better cell internalisation, the significantly higher total cell-associated activity of [^111^In]In-BQ7859 seems to lead to higher activity retention in PSMA-expressing tissues, as indicated by the in vivo results. Even though not statistically significant (*p* = 0.078), the same trend for the higher retention of [^111^In]In-BQ7859 was seen in the tumour, where the decrease in tumour uptake between 1 h and 24 h pi. appeared to be lower for [^111^In]In-BQ7859 when compared with [^111^In]In-BQ7857 (Figure 10A). Another important feature of imaging agents are high T/O ratios, as they are directly related to a higher imaging contrast, and therefore an increased possibility of detecting metastases and small lesions. When comparing the T/O ratios at 24 h pi., [^111^In]In-BQ7859 demonstrated a significantly higher T/blood ratio when compared with [^111^In]In-BQ7857 (Figure 10B). The two-times higher T/blood ratio for [^111^In]In-BQ7859 should lead to an improved signal over background and thus, allow for the detection of smaller lesions [29]. Based on these findings, it appears that the substitution of tranexamic acid for L-tyrosine improves the targeting properties, ultimately promoting [^111^In]In-BQ7859 as a promising imaging agent.

Biological systems are more complex than single properties themselves, making it important to consider more than just the in vitro affinity. Especially for imaging agents, consideration should include properties such as their uptake and clearance from healthy tissue and non-targeting organs, as well as high tumour retention. High activity uptake in the kidneys as observed for [^111^In]In-BQ7859 is typical for PSMA-targeting agents and could complicate the detection of small lesions in the lumbar area. However, the published clinical data for imaging of PCa using [^68^Ga]Ga-PSMA demonstrated that lymph node lesions were visualized while the SUVkidney/SUVtumour ratio was ~3 [19]. Moreover, it has been demonstrated that a high renal excretion of [^177^Lu]Lu-PSMA-617 for radiotherapy (~50% IA) resulted in an acceptable accumulated dose in the kidneys while reaching the limiting doses for the salivary glands [30]. In this study, [^111^In]In-BQ7859 and [^68^Ga]Ga/[^177^Lu]Lu-PSMA-617 were not compared head-to-head in the same system. However, the affinity, internalisation, and biodistribution profile at 1 h pi. of [^111^In]In-BQ7859 were similar to the data reported for PSMA-617 [23]. It is important to take into account that the chemical structure of PSMA-617 differs from BQ7857 and BQ7859 in more aspects than just the linker (Figure 1 and Figure 2). This could make it more complicated to specifically evaluate the effect of the linker when comparing the compounds in this study with the literature data of PSMA-617. Based on this, the compound BQ7857 was included in this study to more easily evaluate the relationship between the linker structure and the targeting properties. The need for the continued investigation of PSMA-binding radioligands is necessary, and hopefully, this study can contribute to the understanding of the relationship between the molecular structure and targeting properties. However, the findings reported herein may also apply to the progress of other compounds containing PSMA-binding domains, such as heterodimeric radioligands targeting PSMA and gastrin-releasing peptide receptor (GRPR). Due to a heterogenic expression of the two targets, some patients may benefit from a heterodimeric tracer with the ability to bind both PSMA and GRPR [31,32]. Therefore, this work may also add value to the understanding and improvement of the PSMA-binding part in such compounds.

In this study, the crystal structure of PSMA and structure-based design were used to generate possible structural linker modification based on the linker structure of PSMA-617. Motivations were focused specifically towards improving the targeting properties. Results suggest that 2-napththyl-L-alanine is more beneficial than tranexamic acid, L-phenylalanine, L-tyrosine, and L-(4-bromo)phenylalanine for promoting interactions with the S1 hydrophobic pocket. Both the size and lipophilicity of the naphthylic structure appear to be crucial to this result. For analogues in SET 2, the computational modelling suggested that substituents could bind into subpocket 2. However, since no large difference in binding affinity could be observed for compounds [^111^In]In-BQ7857-62, this might indicate that the substituents could not interact with Phe546, Trp541, and Arg463, or that their interactions did not influence the affinity. Nevertheless, it was demonstrated that other substituents are accepted in place of the tranexamic acid in the linker which could serve as a synthetically convenient position to fine tune the overall pharmacokinetic properties of the compounds. Of the 11 PSMA-binding radioligands, [^111^In]In-BQ7859, which consists of a linker structure derived from 2-napththyl-L-alanine and L-tyrosine, was shown to possess the most beneficial properties overall. When compared with [^111^In]In-BQ7857, which consists of a linker structure of 2-napththyl-L-alanine and tranexamic acid, [^111^In]In-BQ7859 demonstrated higher cell-associated activity and binding retention to PSMA, as well as an increased T/blood ratio. These results highlight BQ7859 as a promising PSMA-binding radioligand for theranostic applications towards PCa.

## Figures and Tables

**Table 1:** Radiochemical yield, the release of free indium-111 after EDTA-challenge for 3 h, and the K_D_-value for compound [^111^In]In-BQ7837-41 (SET 1). Measurements were done in triplicate. * the labelled compound was challenged with 1000-fold excess of EDTA.

	Compound	Radiochemical Yield (%)	Release after 3 h (%) *	K_D_ (nM)
SET 1	[^111^In]In-BQ7837	98.7 ± 0.4	3.1 ± 0.3	3130
	[^111^In]In-BQ7838	99.0 ± 0.3	1.6 ± 0.2	7520
	[^111^In]In-BQ7839	99.0 ± 0.4	1.5 ± 0.5	7500
	[^111^In]In-BQ7840	98.7 ± 0.4	3.4 ± 0.2	4.39
	[^111^In]In-BQ7841	98.9 ± 0.4	1.5 ± 0.2	147

**Table 2:** Radiochemical yield, the release of free indium-111 after EDTA challenge for 3 h, K_D_-value, and LogD for the compound [^111^In]In-BQ7857-62 (SET 2). Measurements were done in triplicate. * the labelled compound was challenged with 1000-fold excess of EDTA.

	Compound	Radiochemical Yield (%)	Release after 3 h (%) *	K_D_ (nM)	LogD
SET 2	[^111^In]In-BQ7857	98.8 ± 0.5	5.6 ± 1.1	2.67 ± 0.4	−2.97
	[^111^In]In-BQ7858	98.4 ± 0.4	7.0 ± 0.4	9.46 ± 1.3	−2.45
	[^111^In]In-BQ7859	99.1 ± 0.4	3.1 ± 0.6	6.53 ± 0.9	−3.03
	[^111^In]In-BQ7860	99.4 ± 0.4	3.6 ± 0.8	16.5 ± 4.6	−1.42
	[^111^In]In-BQ7861	98.4 ± 0.6	2.8 ± 0.3	12.4 ± 5.0	−1.59
	[^111^In]In-BQ7862	99.3 ± 0.3	1.3 ± 0.2	7.04 ± 1.1	

## Data Availability

The data generated during the current study are available from the corresponding author upon reasonable request.

## Supplementary Materials

The following are available online at https://www.mdpi.com/article/10.3390/pharmaceutics14051098/s1, Figure S1: Overlay of the PSMA-1027 X-ray structure and the suggested binding mode of BQ7857-61, Figures S2–S12: Chemical structure and MS-chromatogram of BQ7837-41 and BQ7857-62, and radio-HPLC chromatogram of [^111^In]In-BQ7837-41 and [^111^In]In-BQ7857-62, Table S1: In vivo biodistribution of [^111^In]In-BQ7857-61 (40 pmol/animal, 30 kBq) in BALB/c nu/nu PC3-pip xenografted tumour-bearing mice at 1 h pi. Uptake of activity was calculated as percent injected dose per tissue weight (% ID/g). Data are presented as average ± standard deviation, Table S2: In vivo biodistribution of [^111^In]In-BQ7857 and [^111^In]In-BQ7859 (40 pmol/animal, 30 kBq) in BALB/c nu/nu PC3-pip xenografted tumour-bearing mice at 24 h pi. To the left; Uptake of activity was calculated as percent injected dose per tissue weight (% ID/g), to the right; tumour-to-organ ratios at 24 h pi. Data are presented as average ± standard deviation.

## Appendix A

### Appendix A.1. General Information

#### Appendix A.1.1. Instruments and Equipment

Analytical high-performance liquid chromatography (HPLC) was performed on a Dionex UltiMate 3000 HPLC system with a Bruker amazon SL ion trap mass spectrometer and detection by UV (diode array detector, 214, 254, and 280 nm) and electrospray ionization (ESI) MS using a Penomenex Kinetex C18 column (50 × 3.0 mm, 2.6 µm particle size, 100 Å pore size) with gradients of H_2_O/CH_3_CN/0.05% HCOOH as mobile phase at a flow rate of 1.5 mL/min. Preparative reversed-phase high-performance liquid chromatography (RP-HPLC) was performed by UV-trigged (214 nm) fraction collection with a Glison HPLC system using a Machery-nagel NUCLEODUR C18 HTec column (21 × 125 mm, particle size 5 µm) and H_2_O/CH_3_CN/0.1% TFA as mobile phase at a flow rate of 15 mL/min. Radio HPLC was performed on a Hitachi Chromaster HPLC system using a Luna C18 column (5 µm, 100 Å, 150 × 4.6 mm from Phenomenex, Værløse, Denmark) with gradients of 5–10% 0.1% CH_3_CN (0–15 min), 70–95% 0.1% CH_3_CN (15–17 min), and 95% 0.1% CH_3_CN (19–20 min). Activity content was measured using an automated gamma counter (3-inch NaI(Tl) detector, 2480 Wizard2, PerkinElmer). Statistical analysis was performed by unpaired, two-tailed t-test using GraphPad Prism 8 for Windows (GraphPad Software Inc, San Diego, CA, USA), *p*-values < 0.05 were considered statistically significant.

#### Appendix A.1.2. Chemicals and Solvents

All chemicals and solvents were purchased from Sigma Aldrich, Fisher Scientific, and VWR, and used without further purification if nothing else stated. NOTAbis(*t*Bu)ester was purchased from CheMatech, France; Fmoc Rink Amide MBHA resin (loading 0.69 mmol/g) and L-Glu(tBu)-O(*t*Bu) were purchased from Iris Biotech Gmbh (Marktredwitz, Germany); PSMA-11 was purchased from ABX (Radeberg, Germany).

#### Appendix A.1.3. Cell-lines

In vitro experiments were performed using the PSMA-expressing isogenic human prostate carcinoma PC3-pip cell line obtained from Dr Warren Heston, Cleaveland Clinic. The PC3-pip cell line was cultured every other week in Rosewell Park Memorial Institute (RPMI)-1640 media (BioWest, France) supplemented with 20% fetal bovine serum, 1% penicillin-streptomycin (100 IU/mL penicillin and 100 µg/mL streptomycin), and 1% 2 nM L-glutamine (all from Biochrom AG, Germany), and every other week in the same media as mention above but with additional supplementation of puromycin (20 µg/mL) (Sigma Aldrich, Steinheim, Germany). All cells were incubated at 37 °C in 5% carbon dioxide gas.

#### Appendix A.1.4. In Vivo model

In vivo experiments were carried out using PSMA-expressing tumour-bearing BALB/c nu/nu mice. To establish PC3-pip xenografts, mice were implanted with 5 × 10^6^ PC3-pip cells two weeks before the experiments. The average animal weight was 15.2 ± 1.8 g. All the experiments were planned and performed according to the national legislation on laboratory animals’ protection and the Declaration of Helsinki, and were approved by the Ethics Committee for Animal Research in Uppsala, Sweden. Approval number: 5.8.18-00473/2021. Approved on 26 February 2021.
