# Supplementary material for: Design, Synthesis, and Evaluation of Linker-Optimised PSMA-Targeting Radioligands
Source: Pharmaceutics. 2022 May 20;14(5):1098. doi: 10.3390/pharmaceutics14051098 (PMC9147442; doi:10.3390/pharmaceutics14051098)
Supplement: Supplementary file 1 [file pharmaceutics-14-01098-s001.zip › pharmaceutics-1721651-supplementary.pdf]

Supplementary Material

Design, Synthesis, and Evaluation of Linker Optimised PSMA-targeting Radioligands

Fanny Lundmark [1], Gustav Olanders [1], Sara Sophie Rinne [1], Ayman Abouzayed [1], Anna Orlova [1,2,†], and Ulrika Rosenström [1,†,*]

[1] Department of Medicinal Chemistry, Uppsala University, 751 23 Uppsala, Sweden; fanny.lundmark@ilk.uu.se (F.L.); gustav.olanders@ilk.uu.se (G.O.) sara.rinne@ilk.uu.se (S.S.R.); ayman.abouzayed@ilk.uu.se (A.A.); anna.orlova@ilk.uu.se (A.O.); ulrika.rosenstrom@ilk.uu.se (U.R.)

[2] Science for Life Laboratory, Department of Medicinal Chemistry, Uppsala University, 751 23 Uppsala

* Correspondence: Ulrika.rosenstrom@ilk.uu.se

† Equal contribution

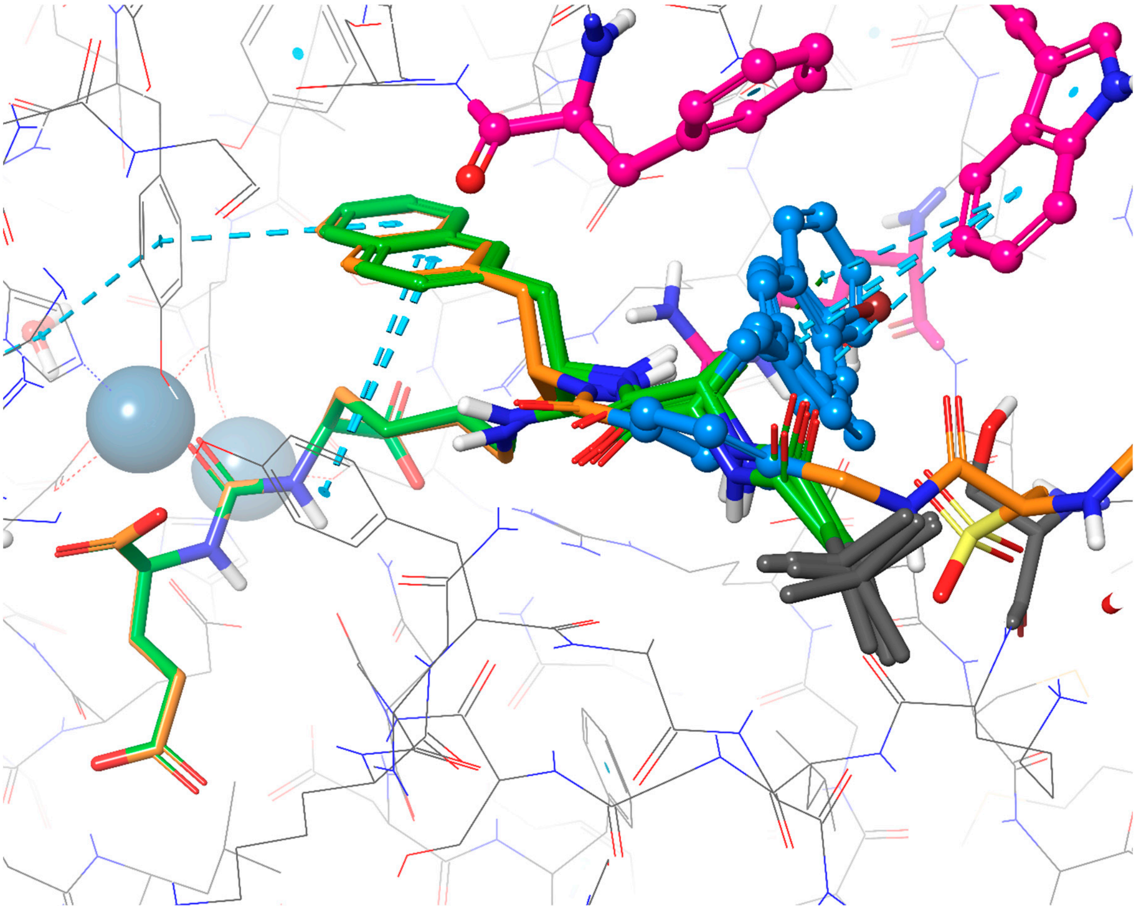

Figure S1. Overlay of the PSMA-1027 X-ray structure (orange) and the suggested binding mode of BQ7857-61 depicted in green with SET 2 changes highlighted in blue. Amino acids Phe546, Trp541, and Arg463 forming subpocket 2 are highlighted in pink. PDB code 5O5U.

Figure S2–S12: Chemical structure and mass-spectroscopy (MS) chromatogram of BQ7837-41 and BQ7857-62, and radio-HPLC chromatogram of [^{111}In]In-BQ7837-41 and [^{111}In]In-BQ7857-62

BQ7837

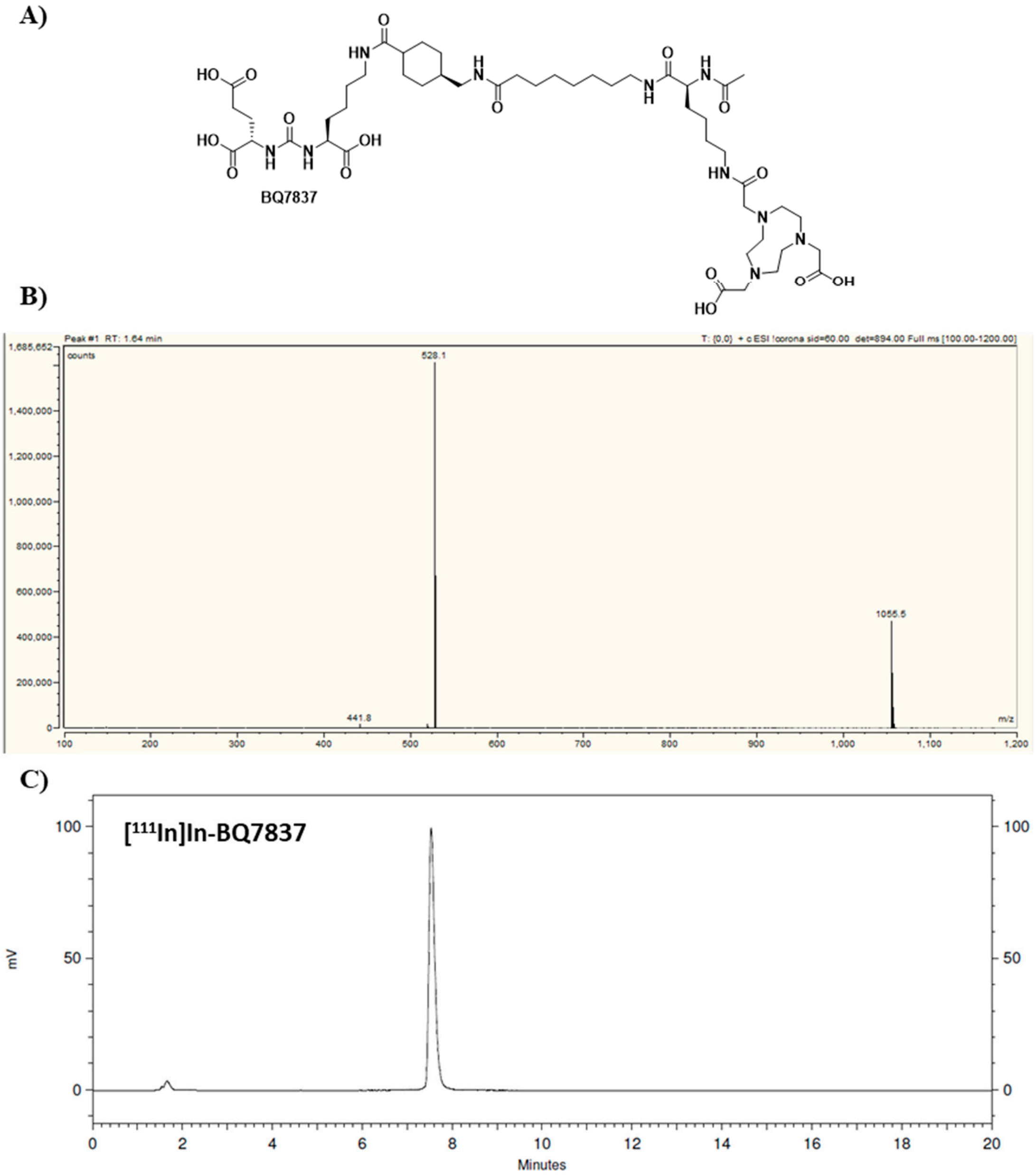

Figure S2. A) Chemical structure of BQ7837, B) MS-chromatogram with observed [M+H]$^{+}$ = 1055.4 and [M+2H]$^{2+}$ = 528.1, C) radio-HPLC chromatogram of [^{111}In]In-BQ7837 demonstrating pure product.

BQ7838

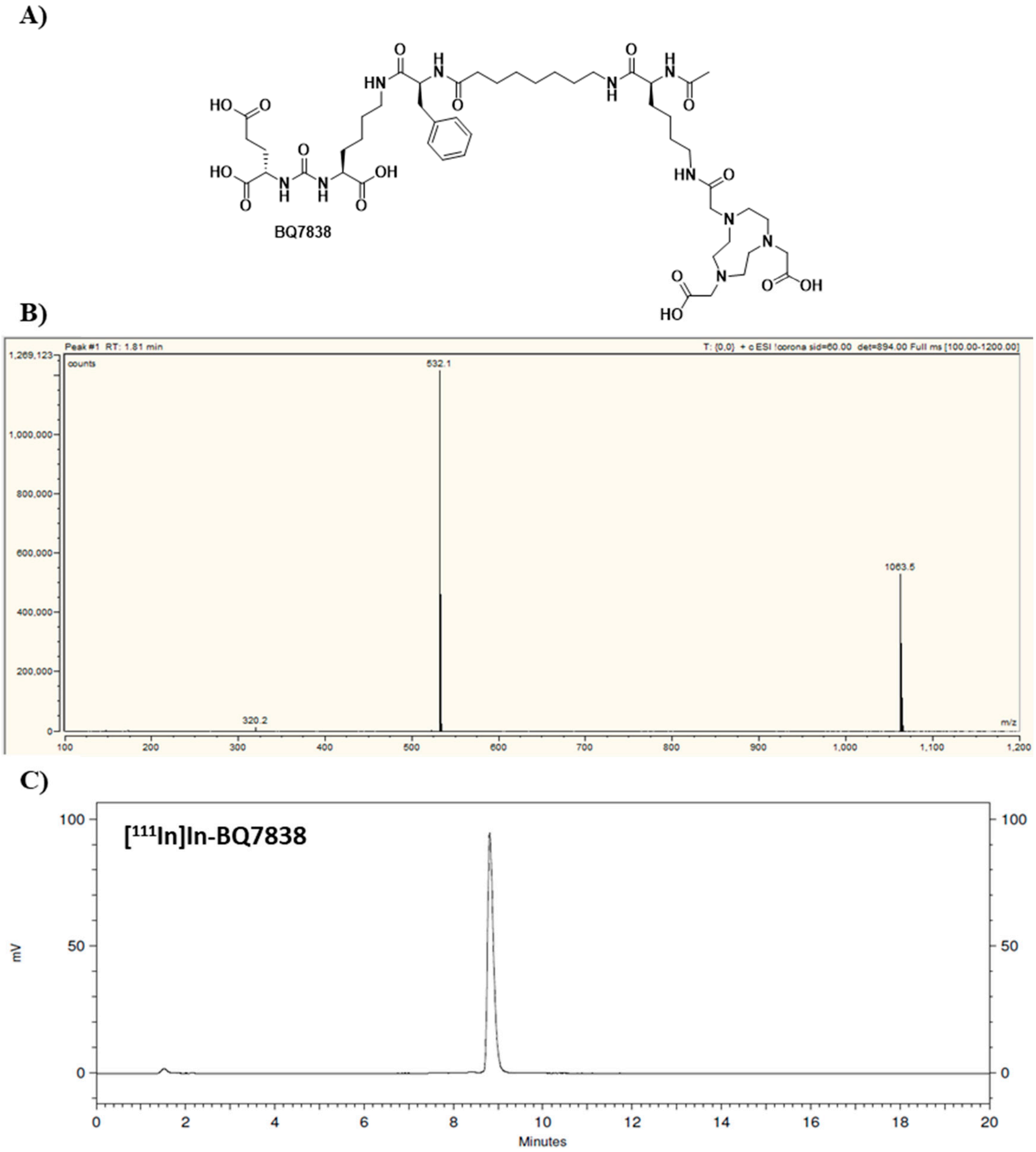

Figure S3. A) Chemical structure of BQ7838, B) MS-chromatogram with observed $[M+H]^+$ = 1063.3 and $[M+2H]^{2+}$ = 532.2, C) radio-HPLC chromatogram of [^{111}In]In-BQ7838 demonstrating pure product.

BQ7839

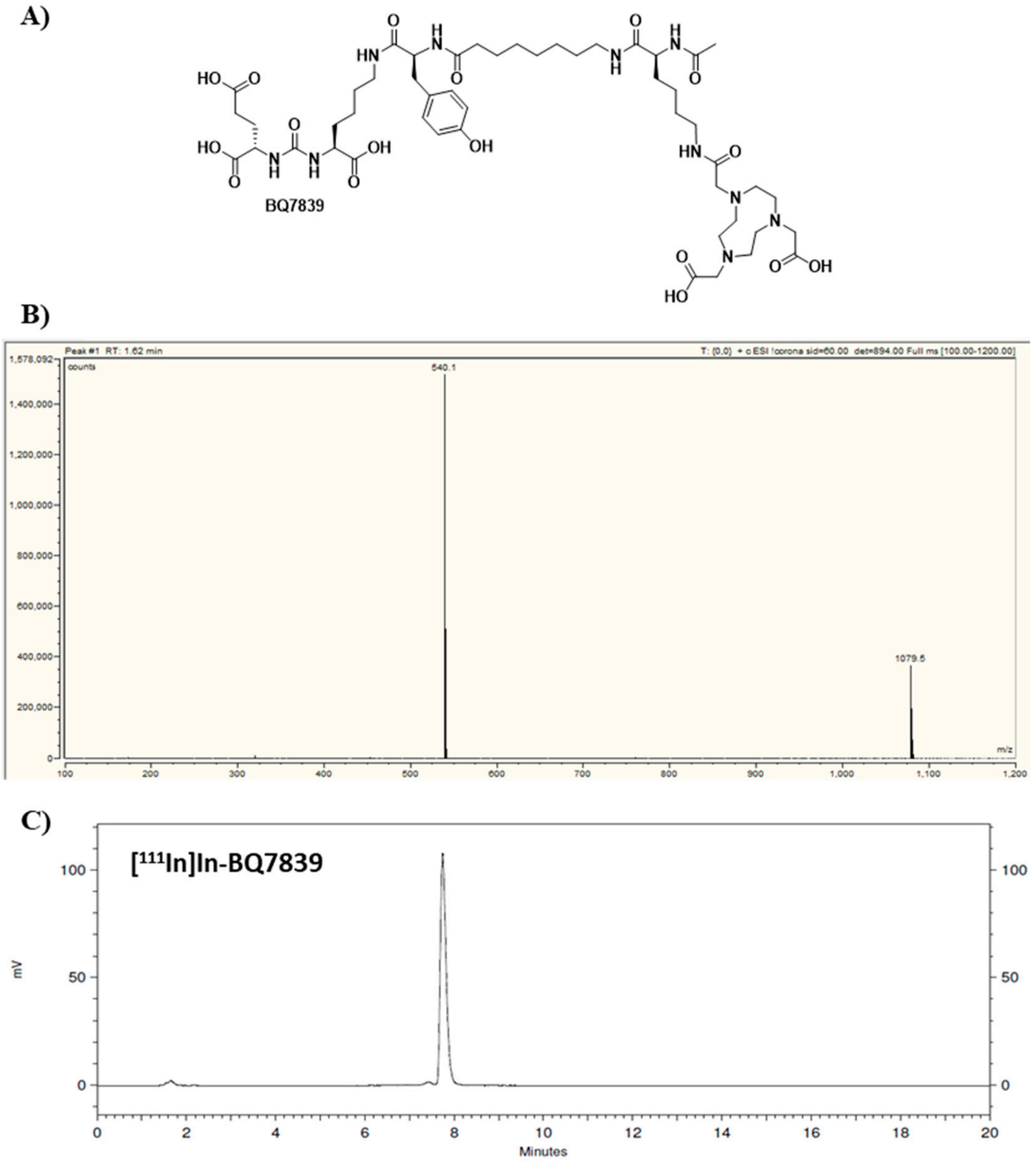

Figure S4. A) Chemical structure of BQ7839, B) MS-chromatogram with observed $[M+H]^{+}$ = 1079.4 and $[M+2H]^{2+}$ = 540.1, C) radio-HPLC chromatogram of [^{111}In]In-BQ7839 demonstrating pure product.

BQ7840

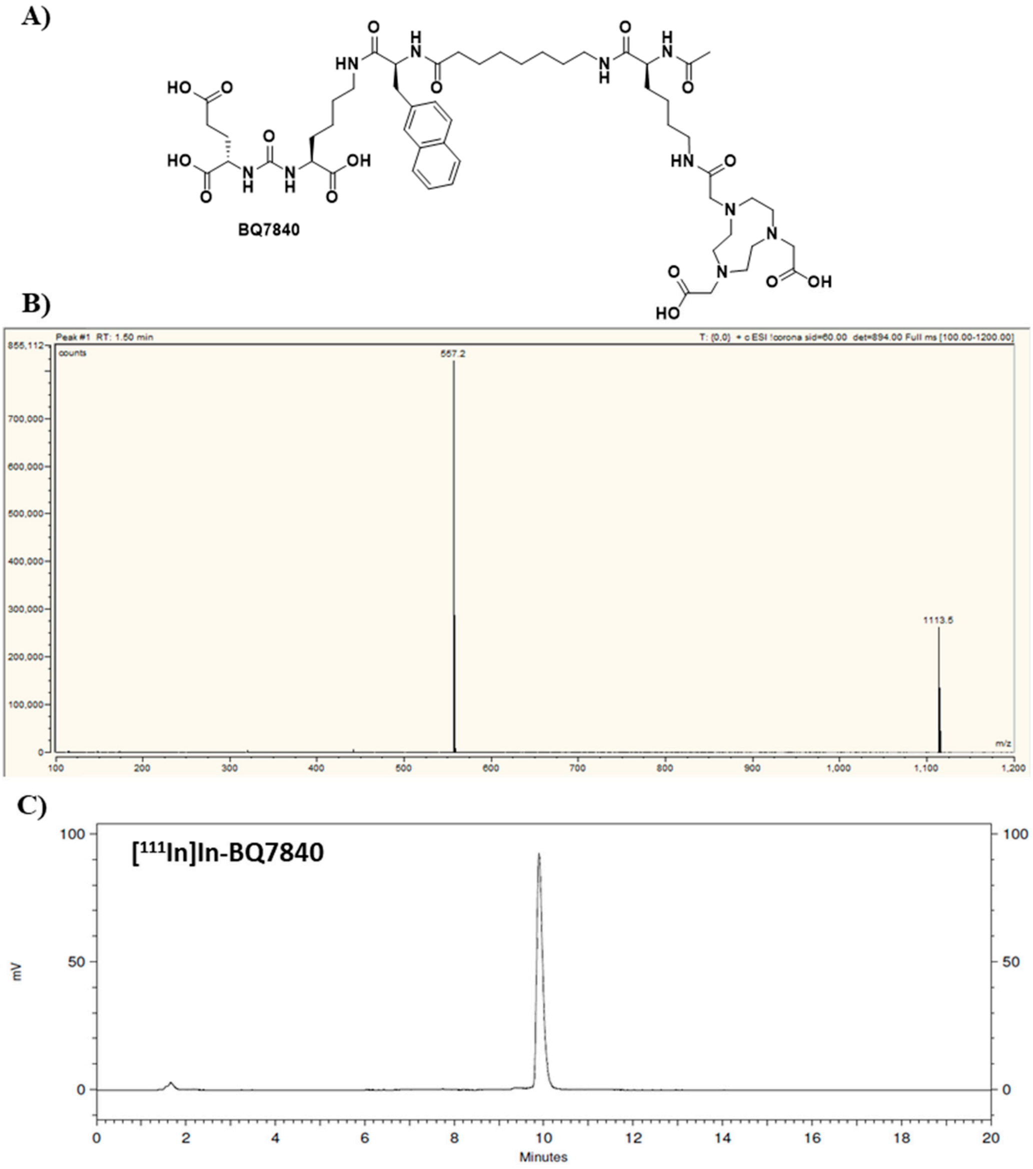

Figure S5. A) Chemical structure of BQ7840, B) MS-chromatogram with observed [M+H]$^{+}$ = 1113.3 and [M+2H]$^{2+}$ = 557.1 , C) radio-HPLC chromatogram of [^{111}In]In-BQ7840 demonstrating pure product

BQ7841

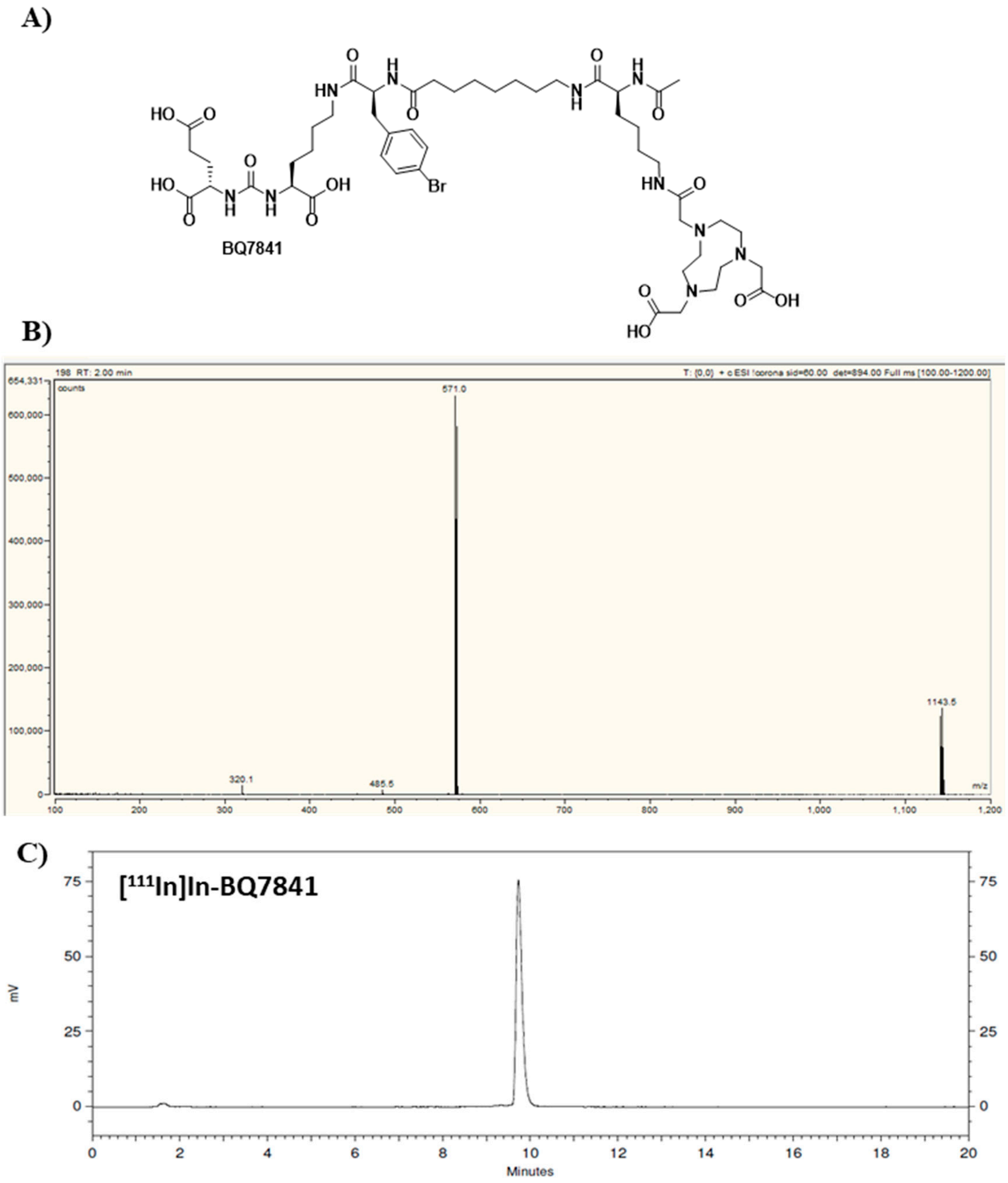

Figure S6. A) Chemical structure of BQ7841, B) MS-chromatogram with observed [M+H]$^+$ = 1141.1/1143.3 and [M+2H]$^{2+}$ = 571.0/572.0 (bromine isotope pattern), C) radio-HPLC chromatogram of [^{111}In]In-BQ7841 demonstrating pure product.

BQ7857

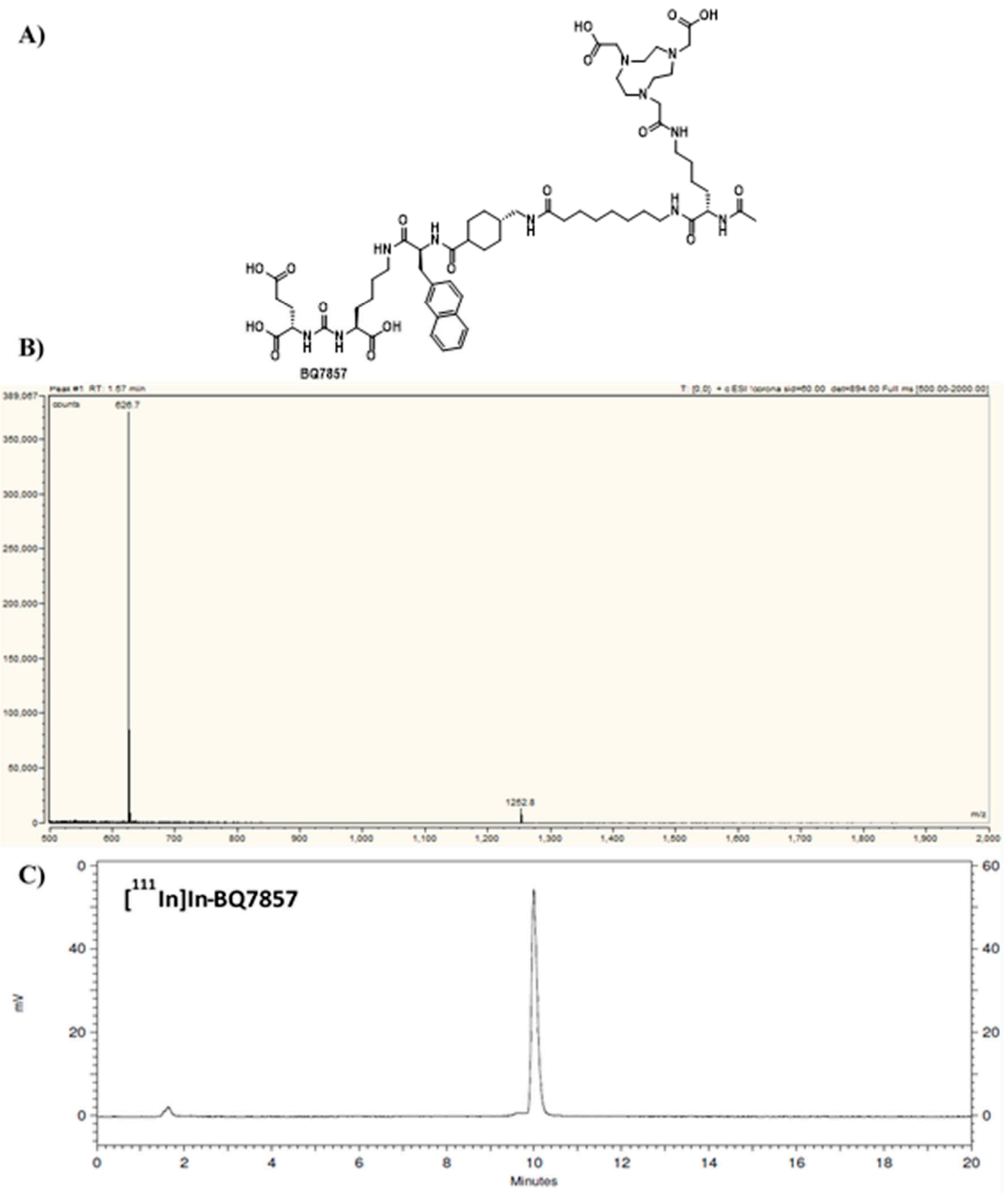

Figure S7. A) Chemical structure of BQ7857, B) MS-chromatogram with observed $[M+H]^+$ = 1252.8.3 and $[M+2H]^{2+}$ = 626.7, C) radio-HPLC chromatogram of $[^{111}In]$In-BQ7857 demonstrating pure product.

BQ7858

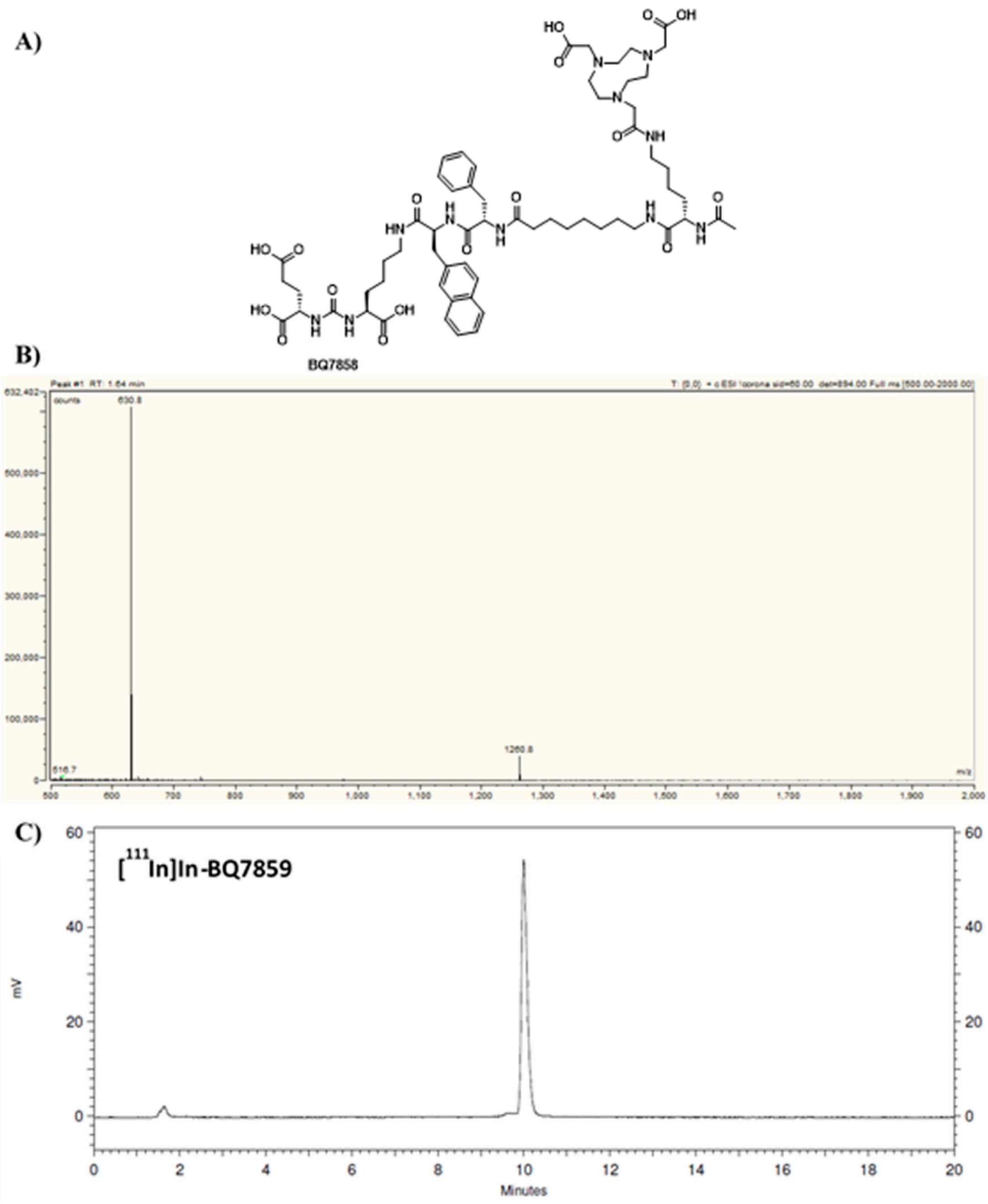

Figure S8. A) Chemical structure of BQ7858, B) MS-chromatogram with observed $[M+H]^+$ = 1260.80 and $[M+2H]^{2+}$ = 630.8, C) radio-HPLC chromatogram of $[^{111}In]$In-BQ7858 demonstrating pure product.

BQ7859

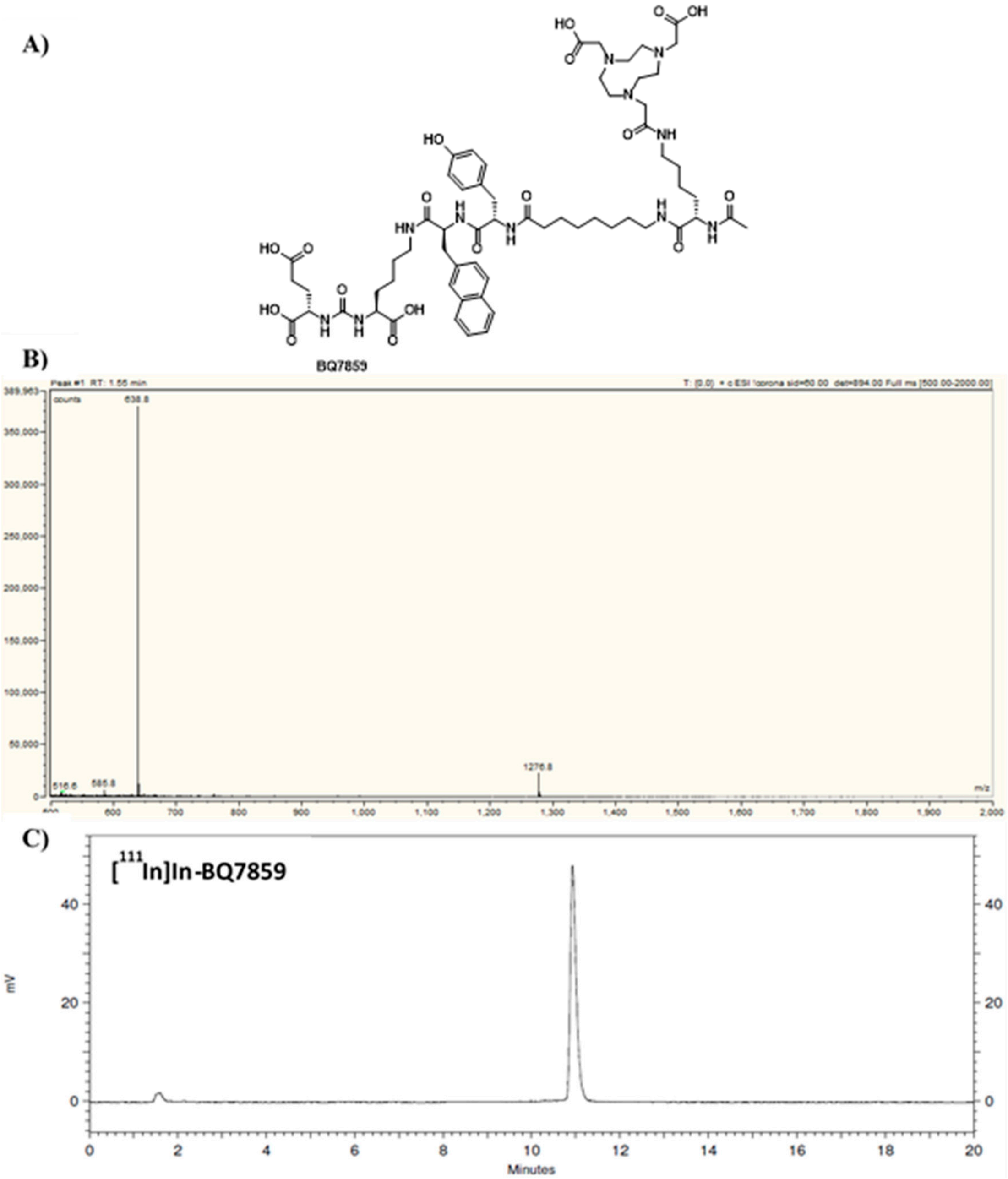

Figure S9. A) Chemical structure of BQ7859, B) MS-chromatogram with observed $[M+H]^+$ = 1276.8 and $[M+2H]^{2+}$ = 638.8, C) radio-HPLC chromatogram of $[^{111}In]In$-BQ7859 demonstrating pure product.

BQ7860

A)

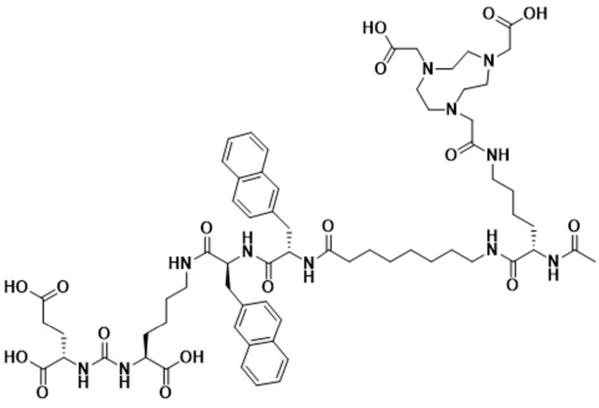

BQ7860

B)

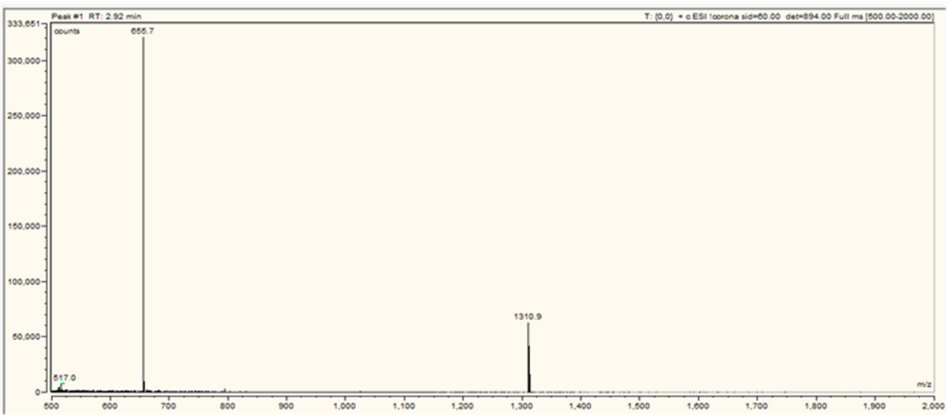

C)

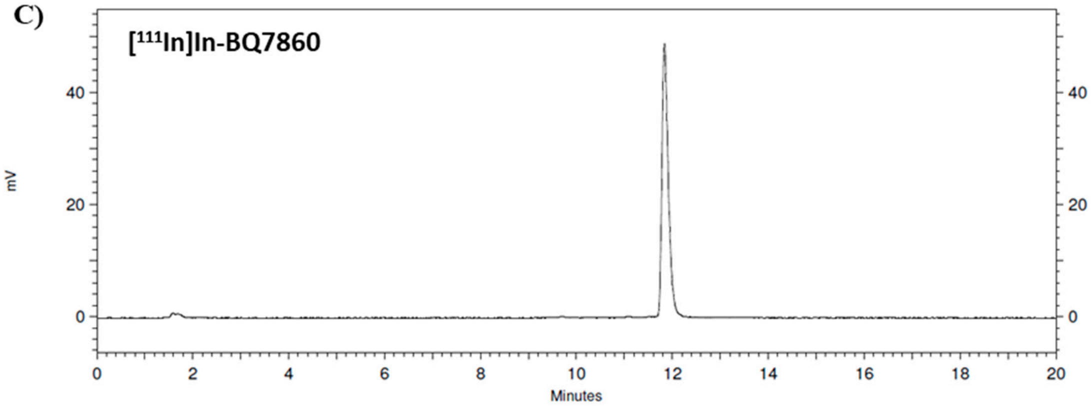

Figure S10. A) Chemical structure of BQ7860, B) MS-chromatogram with observed $[M+H]^+$ = 1310.9 and $[M+2H]^{2+}$ = 656.1, C) radio-HPLC chromatogram of $[^{111}In]In$-BQ7860 demonstrating pure product.

BQ7861

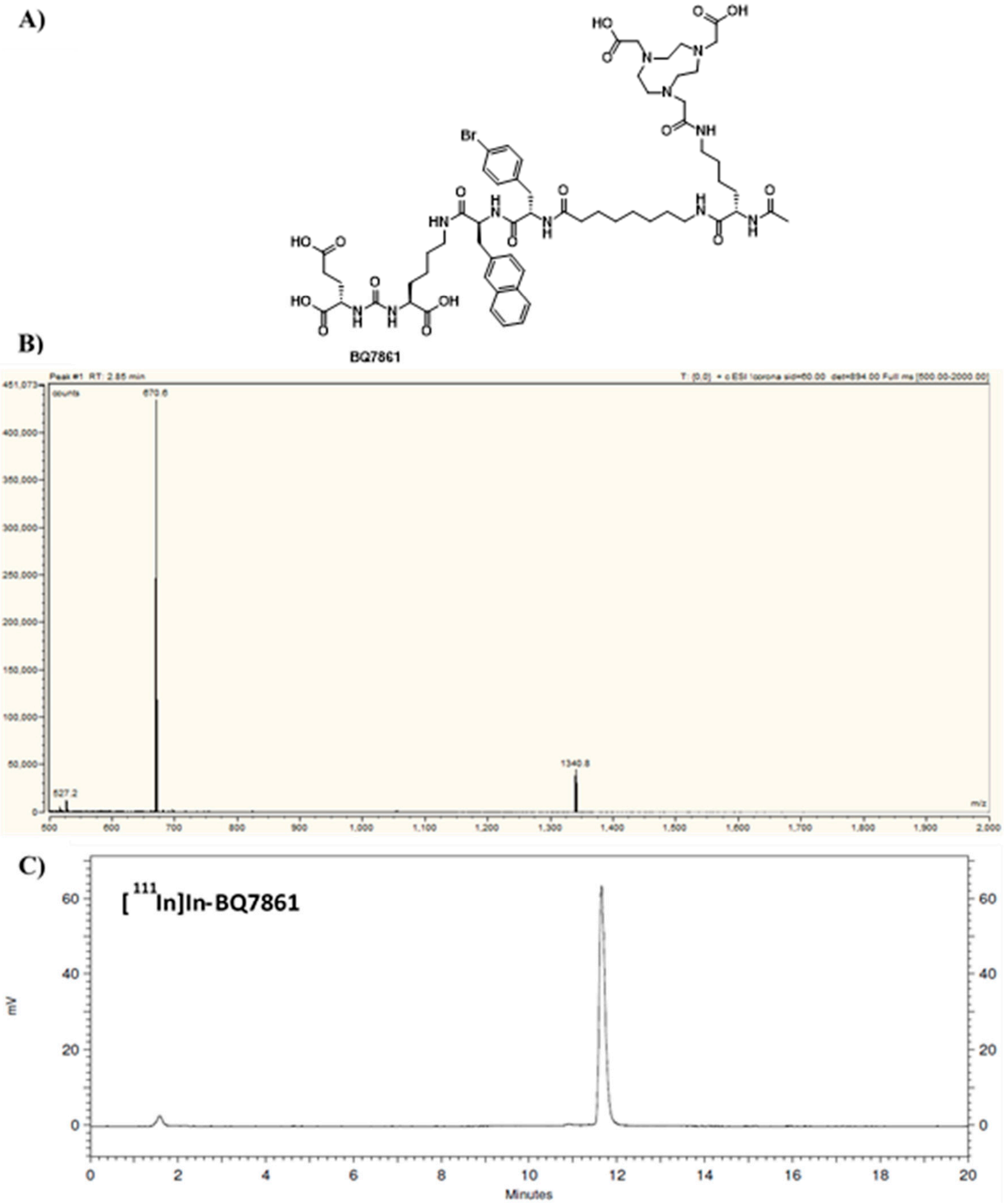

Figure S11. A) Chemical structure of BQ7861, B) MS-chromatogram with observed $[M+H]^{+}$ = 1340.8 and $[M+2H]^{2+}$ = 670.6, C) radio-HPLC chromatogram of $[^{111}In]$In-BQ7861 demonstrating pure product.

BQ7862

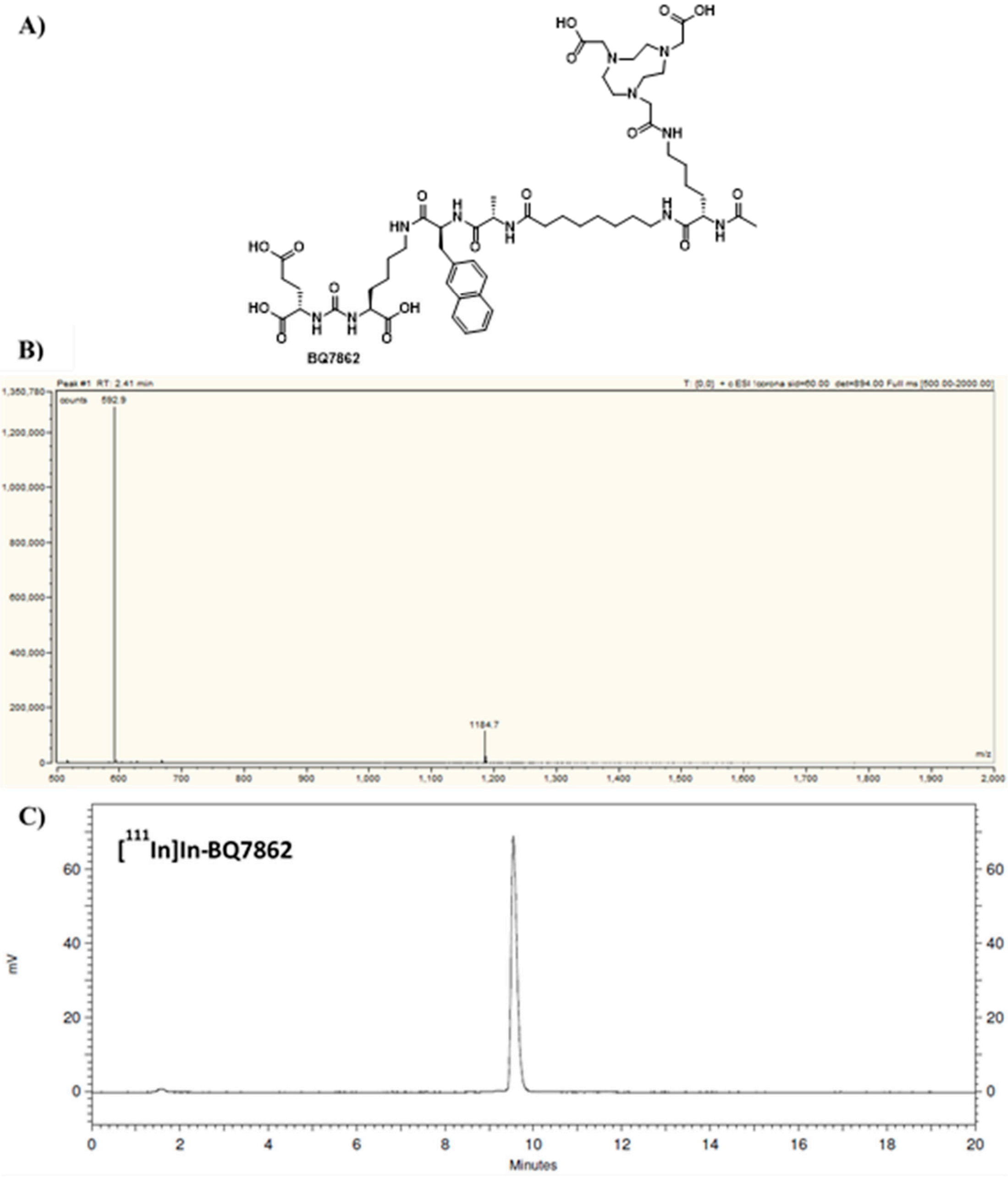

Figure S12. A) Chemical structure of BQ7862, B) MS-chromatogram with observed [M+H]$^+$ = 1184.7 and [M+2H]$^{2+}$ = 592.9, C) radio-HPLC chromatogram of [^{111}In]In-BQ7862 demonstrating pure product.

In vivo biodistribution

Table S1. In vivo biodistribution of [^{111}In]In-BQ7857-61 (40 pmol/animal, 30 kBq) in BALB/c nu/nu PC3-pip xenografted tumour bearing mice at 1 h pi. Uptake of activity was calculated as percent injected dose per tissue weight (% ID/g). Data are presented as average ± standard deviation.

	% ID/g 1h pi.				
**Organ**	**[^{111}In]In-BQ7857**	**[^{111}In]In-BQ7858**	**[^{111}In]In-BQ7859**	**[^{111}In]In-BQ7860**	**[^{111}In]In-BQ7861**
Blood	4.28 ± 0.65	5.04 ± 1.00	3.16 ± 1.73	12.0 ± 1.19	9.18 ± 1.80
Salivary glands	3.18 ± 1.07	3.10 ± 0.56	3.09 ± 1.21	5.83 ± 1.09	3.97 ± 1.84
Lungs	4.32 ± 1.21	5.13 ± 2.47	3.20 ± 1.07	7.76 ± 1.22	5.60 ± 1.02
Liver	1.40 ± 0.30	1.65 ± 0.35	1.38 ± 0.72	5.83 ± 0.73	3.42 ± 0.75
Spleen	13.3 ± 5.27	14.4 ± 1.48	16.3 ± 3.05	22.3 ± 7.63	19.3 ± 8.48
Large intestine	1.16 ± 0.23	1.38 ± 0.28	1.10 ± 0.49	3.40 ± 0.27	1.99 ± 0.31
Kidneys	194.3 ± 31.8	141.5 ± 19.4	182.8 ± 49.5	153.5 ± 27.0	131.0 ± 17.0
Tumour	8.97 ± 2.91	10.6 ± 1.73	10.4 ± 1.78	9.75 ± 1.05	8.98 ± 2.56
Muscle	0.82 ± 2.91	0.95 ± 0.25	0.74 ± 0.33	1.61 ± 0.31	1.24 ± 0.16
Bone	1.27 ± 0.39	1.24 ± 0.20	1.08 ± 0.29	2.54 ± 0.53	2.09 ± 0.20
GI	2.74 ± 0.41	2.67 ± 0.03	1.80 ± 0.43	4.97 ± 1.23	4.20 ± 0.32
Body	14.2 ± 1.14	17.2 ± 3.29	14.1 ± 5.49	32.3 ± 4.29	26.9 ± 6.73

Table S2. In vivo biodistribution of [^{111}In]In-BQ7857 and [^{111}In]In-BQ7859 (40 pmol/animal, 30 kBq) in BALB/c nu/nu PC3-pip xenografted tumour bearing mice at 24 h pi. To the left; Uptake of activity was calculated as percent injected dose per tissue weight (% ID/g), to the right; tumour-to-organ ratios at 24 h pi. Data are presented as average ± standard deviation.

	% ID/g 24h pi.		T/O	
**Organ**	**[^{111}In]In-BQ7857**	**[^{111}In]In-BQ7859**	**[^{111}In]In-BQ7857**	**[^{111}In]In-BQ7859**
Blood	0.041 ± 0.009	0.04 ± 0.02	110.8 ± 1.0	247.2 ± 45.6
Salivary glands	0.13 ± 0.04	0.17 ± 0.06	51.0 ± 28.1	58.3 ± 16.6
Lungs	0.10 ± 0.01	0.13 ± 0.004	57.5 ± 29.7	79.9 ± 15.3
Liver	0.27 ± 0.03	0.37 ± 0.08	21.5 ± 8.73	25.0 ± 7.50
Spleen	0.30 ± 0.04	0.56 ± 0.18	18.7 ± 7.00	16.5 ± 2.14
Large intestine	0.08 ± 0.009	0.07 ± 0.01	74.7 ± 25.4	113.6 ± 19.9
Kidneys	12.5 ± 2.02	30.3 ± 11.6	0.48 ± 0.28	0.32 ± 0.10
Tumour	5.73 ± 2.50	9.00 ± 2.58	1	1
Muscle	0.03 ± 0.005	0.06 ± 0.03	168.5 ± 51.0	167.2 ± 28.3
Bone	0.10 ± 0.01	0.09 ± 0.02	57.0 ± 19.0	102.3 ± 38.3
GI	0.23 ± 0.06	1.78 ± 2.44		
Body	0.96 ± 0.19	1.24 ± 0.52		
